# *Candida* species and oral mycobiota of patients clinically diagnosed with oral thrush

**DOI:** 10.1371/journal.pone.0284043

**Published:** 2023-04-17

**Authors:** Alexandria Sonia Karajacob, Nuramirah Binti Azizan, Anis Rageh Mohammad Al-Maleki, Joanne Pei En Goh, Mun Fai Loke, Hui Min Khor, Gwo Fuang Ho, Sasheela Ponnampalavanar, Sun Tee Tay

**Affiliations:** 1 Department of Medical Microbiology, Faculty of Medicine, Universiti Malaya, Kuala Lumpur, Malaysia; 2 Department of Oral and Maxillofacial Clinical Sciences, Faculty of Dentistry, Universiti Malaya, Kuala Lumpur, Malaysia; 3 Department of Medicine, Faculty of Medicine, University of Malaya Medical Centre, Kuala Lumpur, Malaysia; 4 Department of Clinical Oncology, Faculty of Medicine, University of Malaya Medical Centre, Kuala Lumpur, Malaysia; Ajman University, UNITED ARAB EMIRATES

## Abstract

Overgrowth of *Candida* yeasts in the oral cavity may result in the development of oral thrush in immunocompromised individuals. This study analyzed the diversity and richness of the oral mycobiota of patients clinically diagnosed with oral thrush (OT), follow-up of oral thrush patients after antifungal therapy (AT), and healthy controls (HC). Oral rinse and oral swab samples were collected from 38 OT patients, 21 AT patients, and 41 healthy individuals (HC). Pellet from the oral rinse and oral swab were used for the isolation of oral *Candida* yeasts on Brilliance Candida Agar followed by molecular speciation. ITS1 amplicon sequencing using Illumina MiSeq was performed on DNA extracted from the oral rinse pellet of 16 OT, 7 AT, and 7 HC oral rinse samples. Trimmed sequence data were taxonomically grouped and analyzed using the CLC Microbial Genomics Module workflow. *Candida* yeasts were isolated at significantly higher rates from oral rinse and swab samples of OT (68.4%, p < 0.001) and AT (61.9%, p = 0.012) patients, as compared to HC (26.8%). Predominance of *Candida albicans* specifically, was noted in OT (60.5%, p < 0.001) and AT (42.9%, p = 0.006) vs. HC (9.8%), while non-*albicans Candida* species was dominant in HC. Analysis of oral mycobiota from OT patients showed the presence of 8 phyla, 222 genera, and 309 fungal species. Low alpha diversity (Shannon index, p = 0.006; Chao-1 biased corrected index, p = 0.01), varied beta diversity (Bray-Curtis, p = 0.01986; Jaccard, p = 0.02766; Weighted UniFrac, p = 0.00528), and increased relative abundance of *C*. *albicans* (p = 3.18E-02) was significantly associated with the oral mycobiota of OT vs. HC. This study supported that *C*. *albicans* is the main etiological agent in oral thrush and highlights the association of fungal biodiversity with the pathophysiology of oral thrush.

## Introduction

Oral thrush (oral candidiasis) is an infection resulting from the overgrowth of *Candida* yeasts in the oral cavity [1]. The manifestations of oral candidiasis, most commonly pseudomembranous form, suggests dysfunctional mucosal defense mechanisms [2] in patients with impaired immunity [3]. Oral candidiasis was brought to attention during the human immunodeficiency virus/ acquired immunodeficiency syndrome (HIV/AIDS) epidemic in early 1980s, as it was indicative for the progression of HIV infection to AIDS [4]. The disease is the most prevalent oral manifestation of fungal infections in clinical and dental settings [5, 6], with *Candida albicans* as the primary etiological agent [1]. In recent years, other non-*albicans Candida* (NAC) species have increasingly been reported in cases of oral candidiasis [7], in particular *C*. *glabrata* and *C*. *tropicalis* [8], which poses some challenges in regards to antifungal administration and treatment efficacies [9, 10].

Recent studies have shown that mycobiota plays a role in oral health [11–16]. The pathophysiology of *Candida* yeasts in oral thrush has been extensively researched [14, 17], however the association between the overall host mycobiota diversity and development of oral thrush have not been clearly established. The introduction of Next-generation sequencing (NGS) technology and “omics” [18] led to the identification of a plethora of microorganisms that are non-culturable or missed in routine diagnosis. While most studies focused on the oral microbiome, relatively few studies have characterized the oral mycobiota of patients presenting with oral thrush. The characterization of the human oral mycobiota by Ghannoum et al. [19] has pioneered investigation into oral fungal diseases on a metagenomic level via the analysis of the pan-fungal internal transcribed spacer (ITS) gene. An overlapping of oral fungal genera identified by culture and molecular-based studies (*Alternaria*, *Aspergillus*, *Aureobasidium*, *Candida*, *Cladosporium*, *Cryptococcus*, *Fusarium*, *Penicillium*, *Saccharomyces*) alongside detection of a broader range of fungi via the NGS approach, has helped in shaping our understanding on oral mycobiota diversity and the complex interplay of host-microbial interactions [20].

This study aims to determine the oral mycobiota composition (richness and diversity) in Malaysian patients clinically diagnosed with oral thrush (OT), follow-up oral thrush patients treated with antifungal therapy (AT), and healthy controls (HC), using the conventional culturing method and ITS amplicon sequencing approach. Investigation into the distribution of oral yeast species during oral thrush and after antifungal therapy would provide insight into improved diagnosis and formulation of more targeted treatment strategies. The term oral thrush is used interchangeably with oral candidiasis throughout this paper.

## Materials and methods

### Subject recruitment and clinical diagnosis

This single-center comparative study recruited 38 patients clinically diagnosed with oral thrush (OT) attending medical and dental clinics at University Malaya Medical Center, Malaysia from February 2020 to December 2021. Oral samples were also obtained from 41 healthy control individuals without any clinical signs of oral mucosal diseases (e.g., xerostomia or reduced saliva production). In addition, 21 out of 38 OT patients from dental clinics were able to have a follow-up clinical examination and sampling after two weeks of prescribed treatment with antifungals or instructed to follow daily oral hygiene upkeep (AT). The study was conducted in accordance with the Declaration of Helsinki, and ethical approval was obtained from Universiti Malaya Medical Research Ethics Committee (MRECID no: 2019103–7894). Written informed consent was obtained from all participants and parents of minors prior to sample collection. Due to the coronavirus disease pandemic, the dropout rate for AT patients was ~44.7% (n = 17). DNA samples extracted from oral rinse pellets were selected for NGS based on the ranking of DNA quantity and quality meeting ITS1 amplicon sequencing quality control requirements. A total of 30 samples (9 OT, 7 OT-AT pairs, and 7 HC) were selected for ITS1 amplicon sequencing.

Among the three study groups established (OT, HC, and AT), the inclusion criteria specified recruitment of participants of all ages, with or without underlying disease. Other criteria for all participants included a waiting period of 1 hour following a meal, before the collection of oral samples. The clinical diagnosis of oral thrush was based on visual observations (pseudomembranous, erythematous, angular cheilitis, median rhomboid glossitis and/or denture stomatitis) and positive findings of hyphae and/or spores by Periodic Acid Schiff (PAS) stains from patients’ oral swabs. The exclusion criteria for OT and HC participants were individuals on antifungal therapy, up to six weeks prior to participation in this study. None of the 21 AT patients were found to have oral thrush manifestations during clinical examination upon return. Of the 21 AT patients investigated in this study via culture methods, 15 were treated with Nystatin suspension (1: 500,000 international units, once to three times a day for 14 days; Tystatin oral suspension, Thailand), four had been treated with Miconazole (2% oral gel, once to three times a day for 14 days; Daktarin, India), and one was treated with 2% Miconazole oral gel (three times a day for 14 days; Daktarin, India) and 2% fusidic acid cream (three times a day for 14 days). The remaining one underwent daily oral hygiene upkeep (brushing of gums) instead of antifungal treatment due to age restrictions (1.25 months old). Among the seven AT patients investigated in this study via NGS, six had been treated with Nystatin suspension, while one had been treated with Miconazole. Intra-oral assessments, collection of sociodemographic data of participants (Table 1) were conducted by the respective attending clinicians and recorded on a designated data collection form. Concurrent systemic bacterial infections in this study were reported based on blood culture results that were retrieved from patients’ clinical folders by the attending physicians.

**Table 1:** Demographic information of study participants.

Participant demographics	No. of participants, n = 100 (%)			p-value (OT vs. HC)
	Oral thrush (OT) n = 38 (%)	Healthy control (HC) n = 41 (%)	Follow-up (AT) n = 21 (%)	
**Sex (Female)**	23 (60.5)	20 (48.8)	13 (61.2)	0.368^a^
**Age group (Elderly)**	19 (50.0)	10 (24.4)	10 (47.6)	0.012^b^*
**Age mean years (SD)**	61.5 (20.2)	45.6 (20.7)	58.8 (20.3)	
**Age range (years)**	1.25–86	21–95	1.25–79	
**Ethnicity**				
**Chinese**	22 (57.9)	21 (51.2)	12 (57.1)	0.937^b^
**Indian**	9 (23.7)	12 (29.3)	6 (28.6)	
**Malay**	6 (15.8)	7 (17.1)	3 (14.3)	
**Other**	1 (2.6)	1 (2.4)	0 (0)	
**Smoker (Yes)**	2 (5.3)	8 (19.5)	1 (4.8)	0.057^a^
**Risk of malnutrition** ^c^ **				
**Low**	16 (42.1)	35 (85.4)	11 (52.4)	0.001^b^**
**Medium**	9 (23.7)	2 (4.9)	3 (14.3)	
**High**	8 (21.1)	3 (7.3)	4 (19.0)	
**Antimicrobial wash (Yes)**	7 (18.4)	11 (26.8)	4 (19.0)	0.429^a^
**Denture usage (Yes)** ^d^ **	14 (36.8)	37 (90.2)	9 (42.9)	0.006**
**Topical/inhalational corticosteroid (Yes)**	5 (13.2)	6 (14.6)	4 (19.0)	1.000^a^
**Antibiotic treatment (Yes)** ^e^ *	10 (26.3)	2 (4.9)	0 (0)	0.011^a^**
**Concurrent bacterial infection (Yes)** ^f^ **	7 (18.4)	0 (0)	0 (0)	0.004^a^**
**Cancer (Yes)** ^g^ **	8 (21.1)	0 (0)	1 (4.8)	0.002^a^**
**Chemotherapy (Yes)** *	5 (13.2)	0 (0)	0 (0)	0.022^a^*
**Diabetes (Yes)** ^h^	10 (26.3)	4 (9.8)	4 (19.0)	0.077^a^
**Dyslipidemia (Yes)**	28 (73.7)	5 (12.2)	7 (33.3)	0.153^a^
**HIV (Yes)**	1 (2.6)	0 (0)	0 (0)	0.481^a^
**Hypertension (Yes)** **	13 (34.2)	3 (7.3)	10 (47.6)	0.004^a^**
**Xerostomia (Yes)** **	24 (63.1)	0 (0)	0 (0)	<0.001^a^**
**Type of candidiasis**				
**Pseudomembranous**	19 (50.0)	0 (0)	0 (0)	
**Erythematous**	11 (28.9)	0 (0)	0 (0)	
**Angular cheilitis**	4 (10.5)	0 (0)	0 (0)	
**Median rhomboid glossitis**	3 (7.9)	0 (0)	0 (0)	-
**Denture stomatitis**	3 (7.9)	0 (0)	0 (0)	

^a^Fisher’s exact test

^b^Chi-square test

^c^Six patients preferred not to disclose information related to weight, height and certain clinical conditions

^d^Two OT and AT patients had partial dentures: Upper denture (n = 1) and lower denture (n = 1); One HC participant had partial dentures (upper)

^e^Antibiotic treatment prescribed to oral thrush patients: Ampicillin/Amoxycillin (n = 2), Anti-tuberculosis drug (n = 1), Piperacillin/Tazobactam (n = 3), Augmentin (n = 1), Cloxacillin and Gentamicin (n = 1), Meropenem and Augmentin (n = 1), and Meropenem and Piperacillin/Tazobactam (n = 1). Healthy controls did not disclose the type of antibiotics prescribed

^f^Types of disseminated concurrent bacterial infections: Four bloodstream (one *Staphylococcus aureus*, two *Escherichia coli*, and one undefined Gram-negative bacteria); two respiratory (one *Mycobacterium tuberculosis* and one undefined bacterial species), one bloodstream and genitourinary (*Escherichia coli* and *Klebsiella* sp.)

^g^Hematological (n = 1) and solid organ (n = 7) cancer in OT patients; solid organ cancer (n = 1) in an AT patient

^h^Type of diabetes: Type 1 diabetes (OT, n = 1; HC, n = 0; AT, n = 0), Type 2 diabetes (OT, n = 9; HC, n = 3; AT, n = 4); One HC diabetic patient did not provide the type of diabetes;

*p < 0.05;

** p < 0.01;

-: not applicable

Three age groups of participants were established in accordance with the parameters set by the Department of Statistics Malaysia, i.e., 0 to 14 years for young ages, 15 to 64 years for working ages, and 65 years and above for elderly individuals [21]. Participants with malnutrition risk were categorized into either low (0 points), medium (1 point) or high risk (2 or more points) with the total scores ranging from 0–6 using the Malnutrition Universal Screening Tool, by taking into account the Body mass index (BMI) score (>20 kg/m^2^: 0 points; 18.5–20 kg/m^2^: 1 point; <18.5 kg/m^2^: 2 points), weight loss score (unplanned weight loss of 5%: 0 points; 5–10%: 1 point; >10%: 2 points), and acute disease effect score (0 or 2) [22]. The diagnosis of xerostomia was based on a Clinical Oral Dryness Score, which classified patients into 4 groups with the status of no (0–1), mild (2–4), moderate (5–7) or severe (8–10) xerostomia [23, 24]. This study only categorized OT patients into presence and absence of xerostomia for analysis, while AT patients did not present with xerostomia.

### Sample collection and culture-based identification

Two types of samples, i.e., a sterile cotton swab and an oral rinse sample, were collected from each participant in this study. The swab (Labchem, Malaysia) was used to collect biological samples from the oral mucosae for culturing and Gram staining. Twenty millilitres of oral rinse were collected from each participant as described below: Ten mL of sterile 0.9% sodium chloride mouthwash (Ain Medicare Sdn. Bhd., Malaysia) was offered to each participant to rinse their mouth for one minute, followed by expectoration into a sterile 50 mL centrifuge tube. Each participant repeated the mouth-rinsing procedure twice. Two collection tubes, each with ten mL of oral rinse, were labelled as “oral rinse #1” and “oral rinse #2” for the first and second minute of rinsing.

Each oral swab was cultured onto a Brilliance^™^ Candida Agar (BCA) (Oxoid, UK) plate, a selective differential medium for the rapid isolation and identification of clinically important *Candida* species based on different colony colors for different species. The agar plate was incubated at 37ºC for 48 h in an incubator (Memmert, Germany) as recommended by the manufacturer. The same oral swab was smeared on a sterile glass slide and stained using Gram reagents. Oral rinse #1 samples were centrifuged at 9600 x g in a refrigerated centrifuge (Sigma-Aldrich, USA) at 4ºC for 10 min [14] and the supernatant was discarded. The pellet was resuspended in the oral rinse #2 samples, and divided into three aliquots of 3.33 mL, followed by centrifugation to obtain a pellet. The concentrated pellet from one aliquot was used for this study, while the remaining two aliquots were used for other clinical studies. The concentrated oral rinse pellet was resuspended in 100 microliters (μL) of 0.9% sodium chloride. Ten μL were cultured onto a separate BCA plate, and incubated at 37ºC for 48 h.

The remainder of the pellet (90 μL) was used for ITS1 amplicon sequencing. Concentrated oral rinse was selected for NGS analysis in this study, as it has been demonstrated to be one of the most appropriate sample types to study the mycobiota in the oral cavity [19].

### Molecular identification of *Candida* spp

Oral yeast isolates were identified using PCR-RFLP of the ITS1-5.8S rDNA-ITS2 region, while PCR of the *HWP1* locus was used for *C*. *albicans* and *C*. *dubliniensis* discrimination [25]. Yeast DNA was extracted from all yeast cultures using a Freeze-Thaw DNA extraction method [26]. Each PCR reaction mixture contained 2 μL of DNA template, 4 μL of nuclease-free water, 2 μL of each primer (10 mM; forward and reverse), and 10 μL of ExPrime Taq^™^ Premix 2× (Genet Bio, Korea), which comprised of 1 unit ExPrime Taq^™^ DNA Polymerase, 20 mM Tris-HCl (pH 9.0), 80 mM KCl, 4 mM MgCl_2_, enzyme stabilizer, sediment, loading dye, and 0.5 mM each of dATP, dCTP, dGTP, dTTP), in a total volume of 20 μL. The first pair of primers used for species identification were ITS1 (5’-GTC GTA ACA AGG TTT CCG TAG GTG-3’) and ITS4 (5’-TCC TCC GCT TAT TGA TAT GC-3’) [27]. The second pair of primers used for *C*. *albicans* and *C*. *dubliniensis* discrimination were CR-f (5’-GCT ACC ACT TCA GAA TCA TCA TC-3’) and CR-r (5’-GCA CCT TCA GTC GTA GAG ACG-3’) [28].

All PCR amplification reactions were performed in a Veriti^™^ thermal cycler (Applied Biosystems, USA). The ITS1 PCR amplification conditions were 5 min of initial denaturation at 95ºC for 1 cycle, proceeding with 35 cycles of 95ºC for 1 min, 52ºC for 1 min, 72ºC for 1 min, and one final extension step of 72ºC for 10 min. Restriction fragment length polymorphism (RFLP) of the ITS1-5.8S rDNA-ITS2 region was performed on ITS-amplified PCR products using the FastDigest MspI restriction enzyme (Thermo Scientific, USA). The components in a RFLP reaction were 17 μL of nuclease-free water, 2 μL of 10× FastDigest Green Buffer, 10 μL of PCR product (~ 0.2 μg DNA), and 1 μL of FastDigest enzyme. The contents were incubated at 37ºC on a heat block (Corning, Taiwan) for 5 min, to complete digestion of DNA. The digested DNA was separated by agarose gel electrophoresis. The number of digested bands and fragment sizes were recorded.

Sanger sequencing was performed on the DNA amplicons using forward and reverse primers (ITS1 and ITS4) on the ABI 3730 sequencer (Applied Biosystems, USA). The forward and reverse nucleotide sequences of the yeast isolates were assembled using Bioedit Sequence Alignment Editor version 7.2.5. Manually edited sequences were taxonomically compared to records of deposited strains on the National Centre for Biotechnology Information (NCBI, MD, USA) database (https://blast.ncbi.nlm.nih.gov) using default parameters via the Genbank Basic Local Alignment Search Tool (BLASTn) website.

The *HWP1* PCR thermal cycling conditions included an initial denaturation at 95ºC for 5 mins, followed by 35 cycles of denaturation at 94ºC for 45 sec, annealing at 58ºC for 40 sec, extension at 72ºC for 55 sec and a final extension for 10 min at 72ºC. The PCR product was separated by agarose gel electrophoresis with a band of approximately 700 bp for *C*. *africana*, 941 bp for *C*. *albicans*, and 569 bp for *C*. *dubliniensis*.

### ITS1 amplicon sequencing

#### DNA extraction of concentrated oral rinse samples

DNA from concentrated oral rinse samples (cell pellet) was extracted using the MasterPure^™^ Yeast DNA Purification Kit (EpiCentre, Madison, WI) following manufacturer’s instructions. The genomic DNA was resuspended in 50 μL of TE Buffer and quantified with a nanophotometer (Implen Gmbh, Munich, Germany). The ideal quality control requirements per sample for ITS amplicon sequencing included (i) DNA concentration of ≥ 5 ng/μL, (ii) Total DNA amount of ≥ 500 ng, and (iii) A260/280 Optical Density (OD) purity between 1.8 to 2.0.

The amplification and sequence determination of the fungal ITS gene was performed by Biozeron Biotech Ltd. (Shanghai, PRC). The fungal ITS1 region was amplified using the following conditions: 95°C for 5 min, followed by 29 cycles at 95°C for 30 sec, 55°C for 30 sec, and 72°C for 45 sec, and a final extension at 72°C for 10 min, in a GeneAmp 9700 PCR system (Applied Biosystems, USA). The following primers were used, i.e., ITS1F: 5’—barcode 1—(CTT GGT CAT TTA GAG GAA GTA A)—3’ and ITS2R: 5’- barcode 2—(GCT GCG TTC TTC ATC GAT GC)—3’. Amplicons were extracted from 2% agarose gels and purified using the AxyPrep DNA Gel Extraction Kit (Axygen Biosciences, Union City, CA, USA) according to the manufacturer’s instructions and quantified using QuantiFluor^™^ -ST (Promega, USA). Barcoded ITS1 amplicon libraries were constructed by linking ‘Y’ adapters. Adapter dimers were removed using beads. The concentration of the libraries was determined via PCR amplification. Single-stranded DNA fragments were formed using sodium hydroxide. Sample libraries were pooled in equimolar. The pooled library was paired-end sequenced (2 × 250bp) on the Illumina MiSeq to generate a minimum of 120,000 raw sequencing reads per sample.

#### Filtering of data sequences

Demultiplexing (primer and adapter sequence trimming), quality-filtering and merging of forward and reverse sequences (also known as paired-end) were performed by the sequence provider and executed on QIIME (version 1.9.1) using Trimmomatic and FLASH softwares. Quality-filtering criteria included (i) truncating any site for 300 bp reads that received an average quality score <20 (over a 50 bp sliding window), followed by removal of the truncated reads that were lesser than 50 bp, (ii) precise barcode matching, mismatching of 2 nucleotides in primer matching, discarding reads with ambiguous characters, and (iii) minimum overlap of 10 bp for sequence assembly and exclusion of unassembled reads.

Trimmed sequence data files (fastq format) were imported into CLC Genomics Workbench 21.0.5 (Qiagen, Germany) using the CLC Microbial Genomics Module workflow. The metadata, ITS1 primer sequences, adapter sequences and fungal reference database UNITE v7.2 (https://unite.ut.ee) were simultaneously uploaded.

#### OTU clustering and taxonomical assignment

Trimmed DNA data sequences with 99% sequence similarity were clustered into a single OTU sequence and were taxonomically grouped via the UNITE Classifier against the UNITE fungal database (v7.2). OTU abundance tables were produced, followed by removal of OTUs with low abundance (singletons). The filtered OTU abundance table was selected as input for alignment of OTUs via the multiple sequence alignment approach, known as “MUSCLE”. The filtered OTU alignment was chosen to generate “merge abundance tables”, which was sequentially used for the construction of a Jukes-Canter model neighbor-joining phylogenetic tree (default setting of 100 most abundant OTUs) under the “Maximum Likelihood Phylogeny” tool. The phylogram and merged OTU abundance tables were subsequently used for phylogenetic diversity (alpha and beta diversity) and statistical analysis.

#### Phylogenetic diversity

Alpha diversity evaluations showcased species variations in each population through the Shannon-Weiner (Shannon entropy; Shannon-Weaver) [29] and Chao Index [30]. These approximations were presented in box plots via the rarefaction analysis function with a minimum depth of 1 for 20 points (100 replicates at each point). Beta diversity analysis was executed to estimate differences in species diversity through comparison of two or more populations [31]. The Bray-Curtis (BC), Jaccard (J), Euclidean (E), Unweighted UniFrac (UUF) and Weighted UniFrac (WUF) distance matrix and phylogenetic tree were selected for Principal Coordinate Analysis (PCoA) to explore the relationship between oral thrush status and the diversity of the mycobial community, based on their assignment in a 3-dimensional plot.

#### Statistical analysis

The data collected from the culture and molecular-based investigations were analyzed using Statistical Package for Social Sciences (SPSS) version 24.0 computer software. Categorical variables such as patient-related factors (e.g., age, sex, ethnicity, smoking, risk of malnutrition, antimicrobial wash, denture usage, topical/inhalational corticosteroid, antimicrobial wash, antibiotic treatment, concurrent bacterial infection, cancer, chemotherapy, diabetes, dyslipidaemia, HIV, and hypertension) in OT (n = 38) vs. HC (n = 41) participants were assessed through Fisher’s exact test and/or the Pearson Chi-Square test. Associations of *Candida*, *C*. *albicans*, and NAC isolation rates in OT vs. HC and AT vs. HC groups were also analyzed using Fisher’s exact test and/or the Pearson Chi-Square test, while the McNemar’s test was used to measure associations between paired data (AT vs. OT groups). A two-tailed p-value of < 0.05 was considered statistically significant.

For NGS analysis, nonparametric tests were chosen to evaluate the phylogenetic diversity measures. Alpha diversity measures were analyzed against two statistical tests. The Kruskal-Wallis H test validated statistical difference across three groups (OT, HC, and AT), while the Mann-Whitney U test was the preferred method to identify a statistically significant patient group (p < 0.05) in a group pair (OT vs. HC; AT vs. OT; AT vs. HC). For beta diversity, the permutational multivariate analysis of variants (PERMANOVA) model was applied for comparisons across all groups and groups pairs according to PCoA measures (BC, J, E, UUF, WUF) (Number of permutations: 99, 999), to generate pseudo-f-statistic and Bonferroni-corrected p-values. Host-related factors for OT patients were also analyzed using beta diversity PERMANOVA measures.

Two-tailed independent student t-test was implemented to highlight significant differences (p < 0.05) in the relative abundance and prevalence of species between patient group pairs (OT vs. HC, AT vs. OT, AT vs. HC). For the total number of species detected via NGS, two-tailed independent t-tests were executed for the OT vs. HC and AT vs. HC groups, while a paired sample t-test was executed for the AT vs. OT group, with an alpha value < 0.05. Differential abundance (mean absolute abundance) analysis was performed using a Generalized Linear Model, with the assumption that OTU abundances have Negative Binomial distribution. The Wald test established statistical significance between group pairs (Fold change of either <-2 or >2 and p-value < 0.05), while the likelihood ratio test was implemented across groups for ANOVA-like comparisons to obtain multi-sample Bonferroni corrected p-values.

## Results

### Oral yeasts identification by culturing and molecular methods

#### Clinical evaluation and participant demographics

The sociodemographic data of participants (n = 79) in this study are summarized in Table 1. OT patients were predominantly female (60.5%, n = 23), with an average age of 61.5 (SD: 20.2) years (Table 1). Older age (p = 0.012), greater risk of malnutrition (p = 0.001), antibiotic treatment (p = 0.011), concurrent bacterial infection (p = 0.004), cancer (p = 0.002), chemotherapy (p = 0.022), denture usage (p = 0.006), hypertension (p = 0.004), and xerostomia (p < 0.001) were significantly associated factors with the OT group, as compared to the HC group (Table 1), while variables such as sex (p = 0.368), ethnicity (p = 0.937), antimicrobial wash (p = 0.429), topical/inhalational corticosteroid (p = 1.000), smoking (p = 0.057), diabetes (p = 0.077), dyslipidemia (p = 0.153), and HIV (p = 0.481) were not significantly associated with the clinical diagnosis of oral thrush in this study.

#### Oral yeasts isolation and overall distribution of *Candida* yeasts

Microscopic examination of Gram-stained oral swabs showed the presence of yeast blastoconidia or hyphae in 13.2% (n = 5) of OT patients, while none of the HC and AT participants had fungal elements detected on their oral smears. Of 100 oral specimens cultured on BCA, 81 oral *Candida* isolates were isolated from 50 participants. *Candida* spp. Were isolated most frequently from 26 (68.4%) OT patients followed by 13 (61.9%) AT patients, and 11 (26.8%) healthy individuals. Table 2 depicts the isolation of oral *Candida* yeasts from participants.

**Table 2:** Prevalence of participants with oral yeasts.

Species	No. (%) of participants, n = 100		
	Oral thrush (OT) n = 38 (%)	Healthy control (HC) n = 41 (%)	Follow-up (AT) n = 21 (%)
***Candida* spp.** ^a^	26 (68.4)	11 (26.8)	13 (61.9)
***C*. *albicans***^a^	23 (60.5)	4 (9.8)	9 (42.9)
**Non-*albicans Candida* spp.** ^b^	13 (34.2)	7 (17.1)	6 (28.6)
***C*. *dubliniensis***	5 (13.2)	3 (7.3)	3 (14.3)
***C*. *glabrata***	6 (15.8)	2 (4.9)	2 (9.5)
***C*. *krusei***	1 (2.6)	0 (0)	0 (0)
***C*. *nivariensis***	1 (2.6)	0 (0)	0 (0)
***C*. *parapsilosis* complex**	1 (2.6)	4 (9.8)	3 (14.3)
***C*. *tropicalis***	8 (21.1)	4 (9.8)	2 (9.5)
**Non-*Candida* yeasts**	6 (15.8)	4 (9.8)	2 (9.5)

^a^Presence of *Candida* and *C*. *albicans* were significantly associated with oral thrush status in OT vs. HC groups (p < 0.001) and AT vs. HC groups (p = 0.012; p = 0.006), while it was not for the AT vs. OT groups (p = 1.00; p = 0.375)

^b^Presence of NAC species were not significantly associated with oral thrush status in OT vs. HC groups (p = 0.068), AT vs. HC groups (p = 0.232), and OT vs. AT groups (p = 1.00).

*C*. *albicans* was the most prevalent species in OT (60.5%) and AT (42.9%) groups. However, *C*. *albicans* (9.8%, n = 4) was isolated at a lower rate compared to NAC species (17.1%, n = 7) in the HC group. Notably, *C*. *albicans* was the sole colonizer (only *Candida* species isolated) in 13 (34.2%) OT patients, while a lower incidence was observed in 4 HC (9.8%), and 7 AT participants (33.3%) (Table 3). The presence of *Candida* and *C*. *albicans* isolates (Table 2) were significantly associated with oral thrush status in OT vs. HC (p < 0.001; p < 0.001, respectively). In the absence of oral thrush, *Candida* and *C*. *albicans* were still significantly more prevalent in AT vs. HC groups (p = 0.012; p = 0.006, respectively). However, no significant difference was observed between prevalence of *Candida* (p = 1.00) and *C*. *albicans* (p = 0.375) for AT vs. OT, despite the absence of oral thrush in AT.

**Table 3:** Incidence of single or mixed *Candida* colonization.

Type of species colonisation^a^	No. (%) of participants, n = 100		
	Oral thrush (OT) n = 38 (%)	Healthy control (HC) n = 41 (%)	Follow-up (AT) n = 21 (%)
**Mixed culture of any *Candida* spp.** **Mixed culture of *C*. *albicans* + NAC spp.** **Mixed culture of only NAC spp.**	12 (31.6)	3 (7.3)	4 (19.0)
**Mixed culture of *C*. *albicans* + NAC spp.**	10 (26.3)	0 (0)	2 (9.5)
**Mixed culture of only NAC spp.**	2 (5.3)	3 (7.3)	2 (9.5)
**Single or mixed culture of only NAC spp.**	3 (7.9)	7 (17.1)	4 (19.0)
**Single *C*. *albicans***	13 (34.2)	4 (9.8)	7 (33.3)
**Single *C*. *parapsilosis***	0 (0)	3 (7.3)	1 (4.8)
**Single *C*. *tropicalis***	1 (2.6)	1 (2.4)	1 (4.8)
**CA + CD + CG + CT**	2 (5.3)	0 (0)	0 (0)
**CA + CG + CT**	1 (2.6)	0 (0)	1 (4.8)
**CA + CD + CG**	1 (2.6)	0 (0)	1 (4.8)
**CA + CN + CT**	2.6 (1)	0 (0)	4.8 (1)
**CA + CG**	2 (5.3)	0 (0)	0 (0)
**CA + CP**	1 (2.6)	0 (0)	0 (0)
**CA + CT**	2 (5.3)	0 (0)	0 (0)
**CD + CK**	1 (2.6)	0 (0)	0 (0)
**CD + CP**	0 (0)	0 (0)	2 (9.5)
**CD + CT**	1 (2.6)	0 (0)	0 (0)
**CD + CG + CT**	0 (0)	2 (4.9)	0 (0)
**CD + CP + CT**	0 (0)	1 (2.4)	0 (0)

^a^Abbreviations: +: and; CA: *C*. *albicans*; CD: *C*. *dubliniensis*; CG: *C*. *glabrata*; CK: *C*. *krusei*; CN: *C*. *nivariensis*; CP: *C*. *parapsilosis*; CT: *C*. *tropicalis*; NAC: Non-*albicans Candida*

The most common NAC species in the OT group was *C*. *tropicalis* (21.1%), followed by *C*. *glabrata* (15.8%), which was more commonly featured as compared to the HC and AT groups. In contrast, *C*. *dubliniensis* (14.3%) and *C*. *parapsilosis* (14.3%) were the dominant NAC species in AT, while *C*. *parapsilosis* (9.8%) and *C*. *tropicalis* (9.8%) isolates were the predominating NAC species in HC. Interestingly, *C*. *krusei* (2.6%) and *C*. *nivariensis* (2.6%) were solely cultured from OT patients. In addition, a total of 12 non-*Candida* yeasts (*Cystobasidium*, *Exophilia*, *Magnusiomyces*, *Saccharomyces* and *Trichosporon*) were also isolated on BCA from 6 OT, 4 HC, and 2 AT participants.

Mixed cultures of *Candida* spp. (more than 1 *Candida* species) were observed in 19 participants (OT = 31.6%; HC = 7.3%; AT = 19%; Table 3). Co-isolation of 2, 3, and 4 *Candida* species was obtained from 7, 3 and 2 OT patients, respectively, with *C*. *albicans* most commonly co-isolated with *C*. *glabrata*, and *C*. *tropicalis* (Table 3). A combination of 2 or 3 species were also seen in mixed cultures of HC (7.3%) and AT participants (19%). Amongst all *Candida* species, *C*. *dubliniensis*, *C*. *glabrata*, *C*. *krusei*, and *C*. *nivariensis* were only isolated from mixed cultures, while *C*. *albicans*, *C*. *parapsilosis*, and *C*. *tropicalis* were isolated from both monofungal and mixed fungal cultures.

#### ITS1 amplicon sequence analysis

Sociodemographic factors of 23 participants that were subjected to ITS1 amplicon analysis were summarized in S1 Table. After demultiplexing and filtering, a total of 9,136 predicted OTUs stemmed from 1,998,920 OTU reads (46,110–83,573 per participant) in 30 samples, were representative of 8 phyla, 222 genera and 309 species across the entire study cohort.

#### Alpha and beta diversity of the oral mycobiota

**Fig 1:**
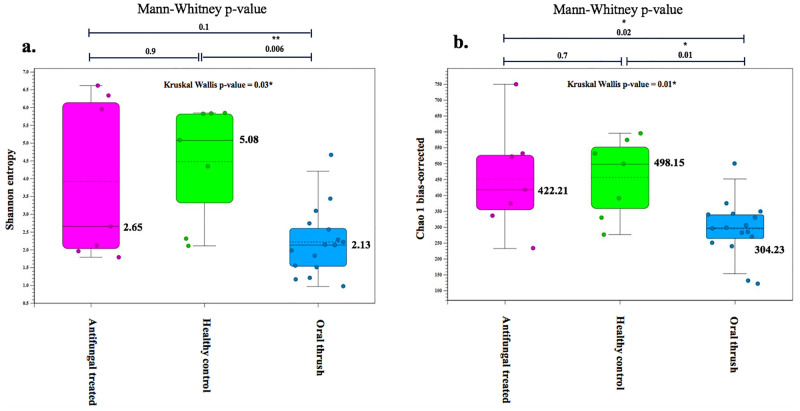
Box plots depicting alpha diversity measures for each study group (AT, HC, and OT). (a) Shannon entropy and (b) Chao-1 biased corrected analysis. The different colors represent the oral thrush status of participants.

Fig 1a and 1b show the Shannon-Weiner and Chao-1 biased corrected indices, which are non-parametric test results for alpha diversity index measures of the oral mycobiota. Significant differences (Kruskal-Wallis test) across three groups of participants (OT, HC, and AT) were established for the Shannon-Wiener index (p = 0.03) and Chao-1 bc estimator (p = 0.01) alpha diversity analysis.

Insight into sample pairs (through the Mann Whitney U test), highlighted significant difference between OT and HC, with reduced diversity (Shannon entropy, p = 0.006) and richness (Chao-1 bc, p = 0.01) in OT patients. Moreover, significantly increased richness was observed in the AT vs. OT patients (Chao-1 bc, p = 0.02). Essentially, fungal community diversity and richness decreased during oral thrush infection (OT), while richness was restored following antifungal treatment (AT). Both alpha diversity measures were not significantly different between AT and HC.

**Fig 2:**
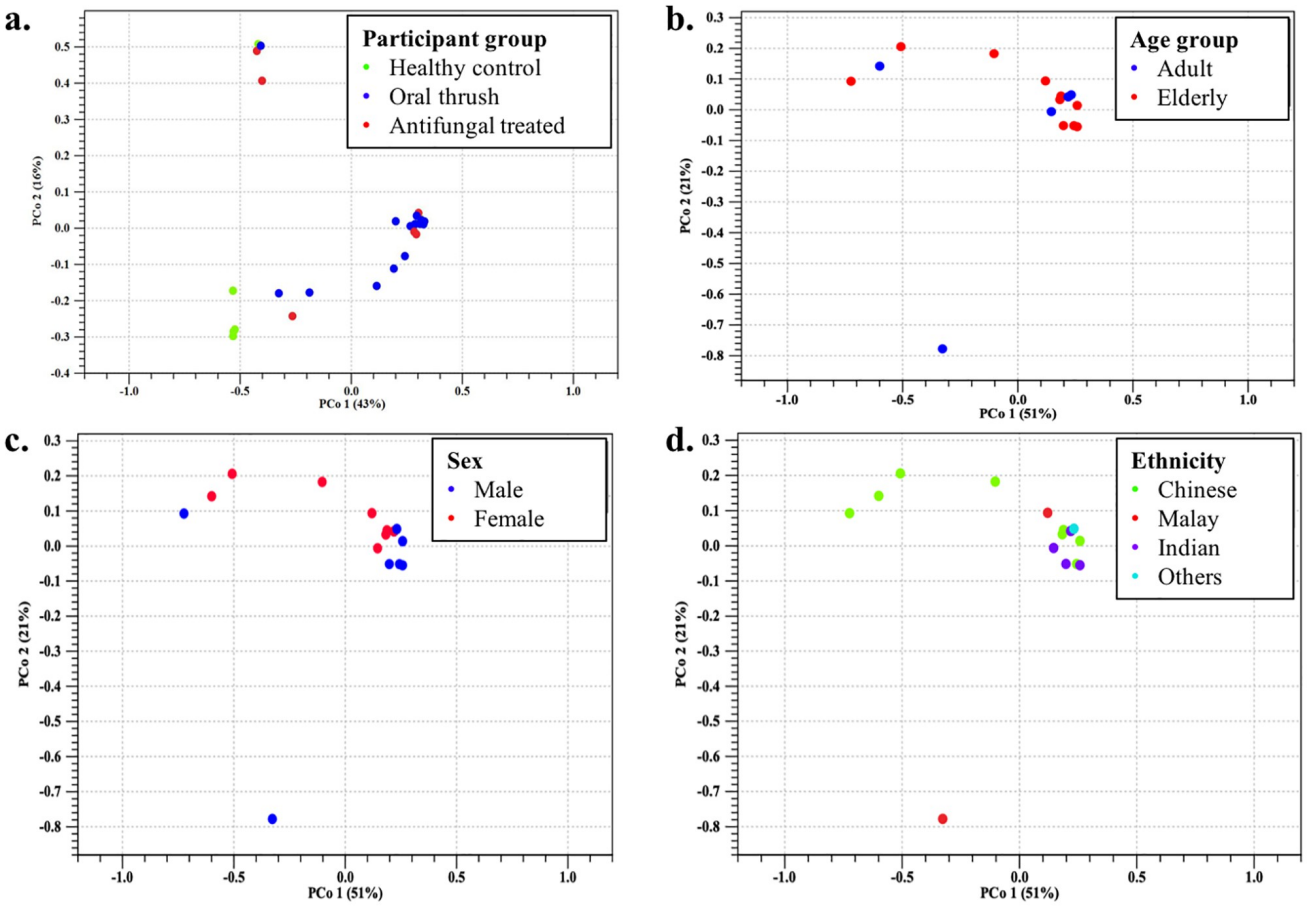
Beta diversity (Bray-Curtis) PCoA plots. The graph highlights the relationship between (a) OT, HC, and AT study groups. Pco1 (16%) and Pco2 (43%) examined the difference in oral mycobiota composition between oral rinse samples, with significant clustering evident in OT vs. HC participants (p = 0.02). Clinical variables such as (b) age, (c) sex, and (d) ethnicity were analyzed for OT patients, with no significant differences observed (p ≥ 0.05).

PCoA ordination analysis displayed significant clustering of the mycobiota between OT, HC, and AT participants for the Bray-Curtis (BC, p = 0.01888; Fig 2a), Jaccard (J, p = 0.03076), Unweighted UniFrac (UUF, p = 0.03624), and Weighted UniFrac (WUF, p = 0.00474) beta diversity measures, excluding the Euclidean (E, p = 0.17721) analysis. The oral mycobiota structure in OT patients showed statistically significant clustering (BC, p = 0.01986; J, p = 0.02766; WUF, p = 0.00528) that was dissimilar to HC individuals, whereas AT patients had widely dispersed oral mycobiota profiling, which also showed significant difference compared to OT patients (UUF, p = 0.04635), but not compared to HC participants.

Differences in the oral mycobiota composition based on host factors were analyzed for OT participants. Factors such as background (age, sex, and ethnicity; Fig 2b–2d), lifestyle (smoking habits and risk of malnutrition), oral health (dentures), type of comorbidity (diabetes, dyslipidemia, hypertension, and xerostomia), and clinical manifestation of oral thrush did not significantly affect the mycological resident distribution of the oral biocompartment in OT patients. While, the sample size was too small for host factors such as antimicrobial wash, topical/inhalational corticosteroid, antibiotic treatment, concurrent bacterial infection, cancer, chemotherapy, and HIV to proceed with PCoA analysis.

#### Genus

*Core oral mycobiota genera*. The core or basal oral mycobiota in this study refers to the fungal distribution based on prevalence (PR) and (relative abundance) AR criteria set by Ghannoum et al. [19], whereby fungal oral residents simultaneously displayed a minimum of 20% PR and 1% (or 1.00E-02) AR. Based on this definition, the core oral mycobiota for the OT group involved two genera, i.e., *Candida* and *Aspergillus* (Table 4).

**Table 4:** Prevalence and relative abundance of fungi at genus level (top 10 most abundant in each patient group; arranged from highest to lowest genera relative abundance in OT patients).

Genus	Prevalence, n (%)			Relative Abundance		
	Oral thrush (OT) n = 16 (%)	Healthy control (HC) n = 7 (%)	Follow-up (AT) n = 7 (%)	Oral thrush (OT)	Healthy control (HC)	Follow-up (AT)
***Candida***	16 (100)	7 (100)	7 (100)	9.39E-01	6.54E-01	7.79E-01
***Aspergillus***	11 (68.75)	4 (57.14)	7 (100)	2.36E-02	7.74E-03	9.64E-03
***Gliocladium***	3 (18.75)	0 (0)	0 (0)	6.37E-03	0	0
***Malassezia***	15 (93.75)	7 (100)	7 (100)	3.79E-03	1.31E-02	2.57E-02
***Peniophora***	5 (31.25)	4 (57.14)	1 (14.29)	3.49E-03	2.51E-05	1.62E-03
***Byssochlamys***	4 (25)	1 (14.29)	2 (28.57)	2.27E-03	2.02E-02	1.04E-02
***Cadophora***	2 (12.5)	0 (0)	0 (0)	1.87E-03	0	0
***Mortierella***	13 (81.25)	7 (100)	7 (100)	1.80E-03	3.15E-02	1.14E-02
***Russula***	1 (6.25)	0 (0)	0 (0)	1.76E-03	0	0
***Neurospora***	5 (31.25)	7 (100)	7 (100)	1.39E-03	4.73E-02	1.26E-02
***Cladosporium***	13 (81.25)	5 (71.43)	6 (85.71)	1.05E-03	7.33E-03	6.15E-03
***Gibellulopsis***	5 (31.25)	4 (57.14)	6 (85.71)	5.08E-04	1.30E-02	4.82E-03
***Myceliophthora***	4 (25)	6 (85.71)	4 (57.14)	3.07E-04	1.51E-02	3.44E-03
***Trichoderma***	2 (12.5)	3 (42.86)	5 (71.43)	1.26E-04	1.72E-02	6.39E-03
***Trichosporon***	6 (37.5)	3 (42.86)	6 (85.71)	1.15E-04	5.20E-02	6.70E-04
***Sampaiozyma***	2 (12.5)	2 (28.57)	5 (71.43)	1.04E-04	1.47E-02	2.55E-02
***Staphylotrichum***	1 (6.25)	1 (14.29)	2 (28.57)	2.15E-05	4.30E-05	6.72E-03
**Others**	16 (100)	7 (100)	7 (100)	1.11E-02	8.68E-02	7.69E-02
***Corallomycetella*** ^a^	1 (6.25)	2 (28.6)	4 (57.14)	3.32E-04	1.13E-02	4.18E-04
***Chaetomium*** ^a^	7 (43.75)	5 (71.4)	7 (100)	8.27E-04	1.10E-02	2.77E-03

^a^*Corallomycetella* and *Chaetomium* are not in the top 10 most relative abundant genera. They are part of the core oral mycobiota in HC individuals

In comparison, the core oral mycobiota of HC comprised of 11 genera, i.e., *Candida*, *Trichosporon*, *Neurospora*, *Mortierella*, *Trichoderma*, *Myceliophthora*, *Sampaiozyma*, *Malassezia*, *Gibellulopsis*, *Corallomycetella*, *and Chaetomium* (Table 4). Meanwhile, the core oral mycobiota in AT, consisted of 6 genera, i.e., *Candida*, *Malassezia*, *Sampaiozyma*, *Neurospora*, *Mortierella*, and *Byssochlamys* (Table 4).

Analysis of the core oral mycobiota highlighted that only *Candida* was found across the three groups. Interestingly, *Aspergillus* was part of the core oral mycobiota, only in OT (Table 4), while *Trichosporon*, *Trichoderma*, *Myceliophthora*, *Gibellulopsis*, *Corallomycetella*, and *Chaetomium* were only evident in HC, and *Byssochlamys* was only observed in AT. The genera *Neurospora*, *Mortierella*, *Sampaiozyma*, and *Malassezia* were simultaneously present in both core oral mycobiota of OT and AT.

#### Species

**Fig 3:**
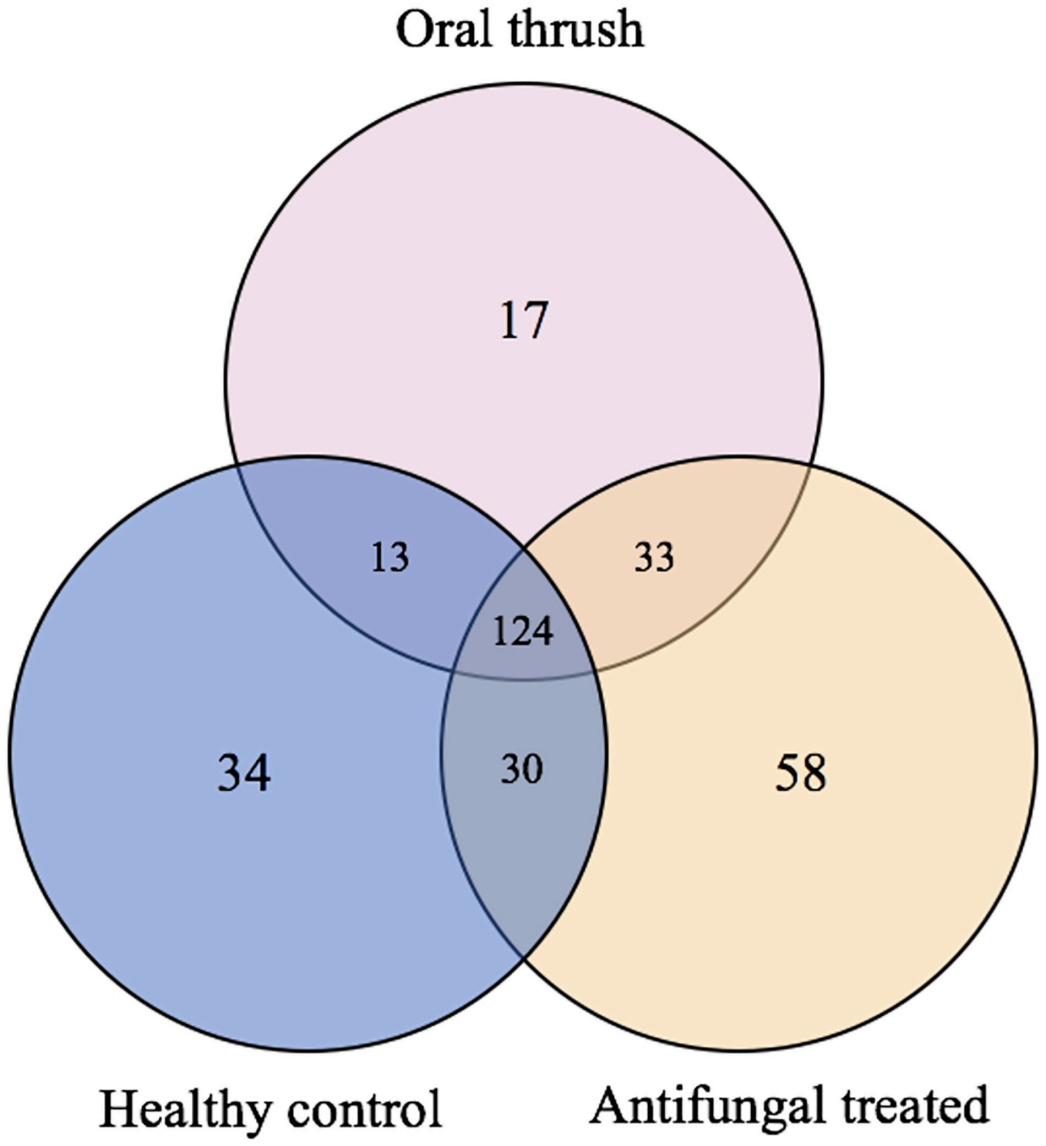
Venn diagram illustrating the number of shared and distinct species in the oral mycobiota of oral thrush, healthy control, and follow-up participant oral rinse samples.

**Fig 4:**
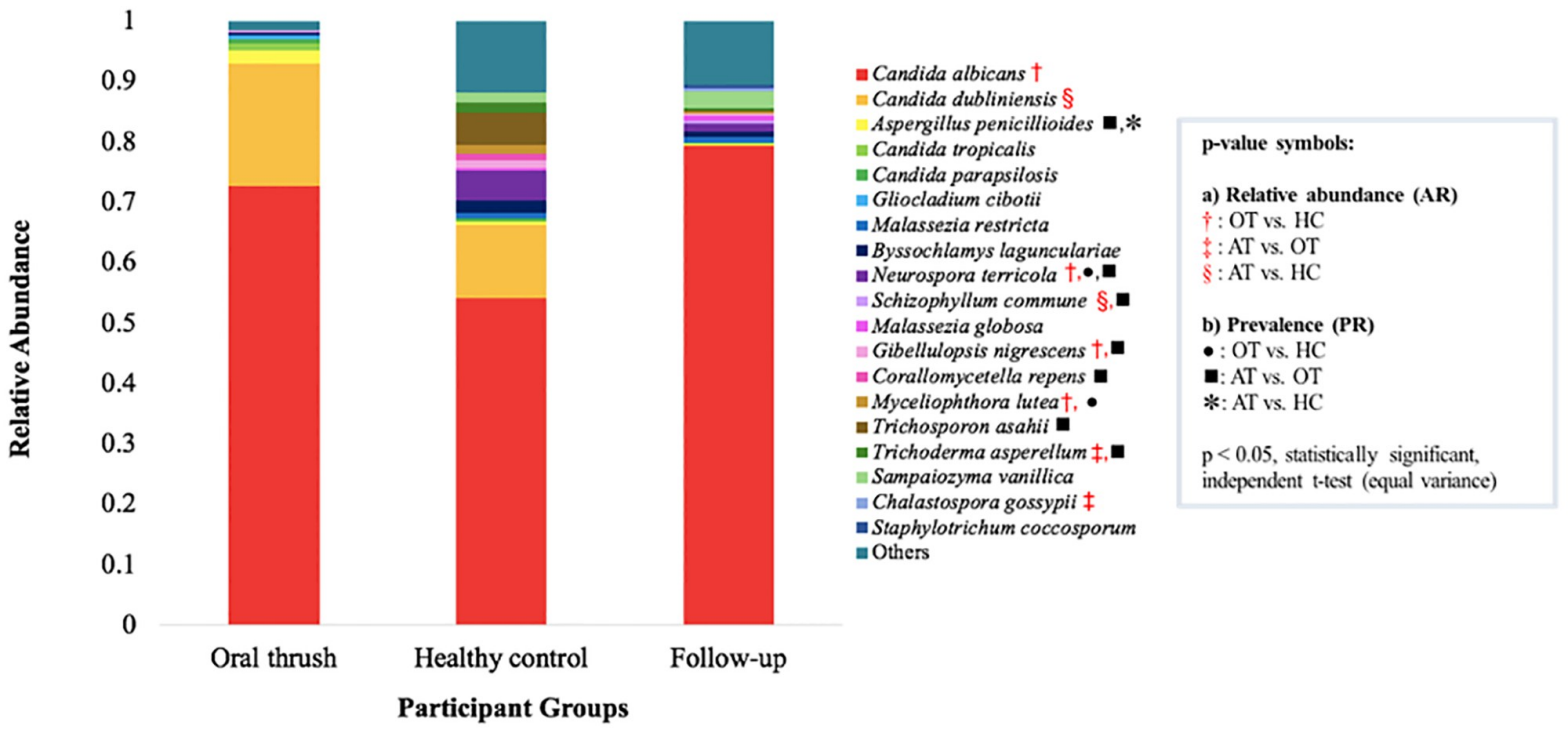
Relative abundance of the top 10 fungal species according to study group (arranged from highest to lowest species relative abundance in OT patients).

*Distinct or shared species in OT*, *HC*, *and AT*. The total number of fungal species detected in this study was 309, with 124 common species across OT, HC, and AT oral rinse samples (Fig 3). The most abundant of the common species (top 5) were *C*. *albicans*, *C*. *dubliniensis*, *Aspergillus penicillioides*, *Neurospora terricola*, and *Trichosporon asahii* (Fig 4; S2 Table). OT had the lowest total number of fungal species annotated (total: 187, range: 9–105) compared to the HC (total: 201, range: 23–96), which was significantly different (p = 0.025), while it was not significantly different (p = 0.076) with AT (total: 245, range: 35–115). As for AT vs. HC, it was also not statistically different (p = 0.213). 17 species were unique to OT, 58 species were unique to AT, and 34 species were unique to HC.

*Core oral mycobiota species in OT*, *HC*, *and AT*. The core oral mycobiota for the OT, consisted of four species, *C*. *albicans*, *C*. *dubliniensis*, *Aspergillus penicillioides*, and *C*. *tropicalis* (Fig 4; S2 Table). In HC, 7 species comprising of *C*. *albicans*, *C*. *dubliniensis*, *Trichosporon asahii*, *Neurospora terricola*, *Sampaiozyma vanillica*, *Myceliophthora lutea*, and *Gibellulopsis nigrescens* were detected (Fig 4; S2 Table). In AT, four species were observed as part of the core oral mycobiota. *C*. *albicans* was similarly seen in the OT and HC, while *Sampaiozyma vanillica*, *Neurospora terricola* were only mirrored in HC. *Byssochlamys laguncularia* was also part of the core oral mycobiota following antifungal treatment, which was not observed in other groups (Fig 4; S2 Table).

*Prevalence and relative abundance of fungal species in OT*, *HC and AT*. The most relative abundant fungal species (top 10) for each study group were presented in Fig 4 (S2 Table). Species with significant ARs for OT vs. HC, AT vs. OT, and AT vs. HC group pairs were reported in S4–S6 Tables. *C*. *albicans*, *C*. *dubliniensis*, *C*. *tropicalis*, and *C*. *parapsilosis* were amongst the top 10 fungal species present in OT. *C*. *albicans* was the predominant species for all three cohorts (PR: 100%; AR ≥ 0.05) with the highest AR in the AT (AR: 7.27E-01; Fig 4). Most importantly, *C*. *albicans* had a significantly higher AR in OT vs. HC (p = 3.18E-02), while it was not significant for the other group pairs (Fig 4).

C. *dubliniensis* was highly prevalent (71.4%—87.5%) across all groups (Fig 4). OT had a higher AR of *C*. *dubliniensis* as compared to the HC, though it was not significant (p = 3.72E-01; Fig 4). Decreased AR of *C*. *dubliniensis* was recorded in the oral mycobiota of participants following antifungal treatment, though it was only significantly lower in AT vs. HC (p = 2.82E-02), and not in AT vs. OT (Fig 4). *C*. *tropicalis* and *C*. *parapsilosis* had the greatest AR in OT, followed by HC, and AT, but these species were not significantly different between group pairs (p ≥ 0.05; Fig 4).

Non-*Candida* core fungi in OT were from the genus *Aspergillus*, with *Aspergillus penicillioides*, having a lower PR (p ≥ 0.05) but increased AR (p ≥ 0.05; Fig 4), compared to the HC and AT groups. Other non-*Candida* core host mycobiota in the HC group with significantly greater AR for HC vs. OT participants included *Neurospora terricola* (AR, p = 7.07E-03; PR, p = 1.18E-03), *Myceliophthora lutea* (PR, p = 5.11E-03; AR, p = 5.42E-03), and *Gibellulopsis nigrescens* (AR, p = 6.51E-03; Fig 4). In the AT group, non-*Candida* core species, was encompassed by *Neurospora terricola* (PR, p = 1.18E-03), *Sampaiozyma vanillica* (PR, p = 3.09E-03), and *Byssochlamys laguncularia* (PR p ≥ 0.05), which were in greater PR for AT vs. OT participants (S5 Table). Amongst species in the top 10 AR of AT patients were *Trichoderma asperellum* (PR, p = 3.09E-03; AR, p = 3.70E-02), *Gibellulopsis nigrescens* (PR, p = 1.47E-02), and *Schizophyllum commune* (PR, p = 3.38E-02) with significantly increased PR and/or AR compared to OT patients (Fig 4).

In this study, *Malassezia restricta* and *Malassezia globosa* were within the top 10 species AR of the OT and AT group, respectively. These species had greater PR and AR in HC and AT participants, as compared to OT patients, though the difference was not significant (p ≥ 0.05; Fig 4).

**Fig 5:**
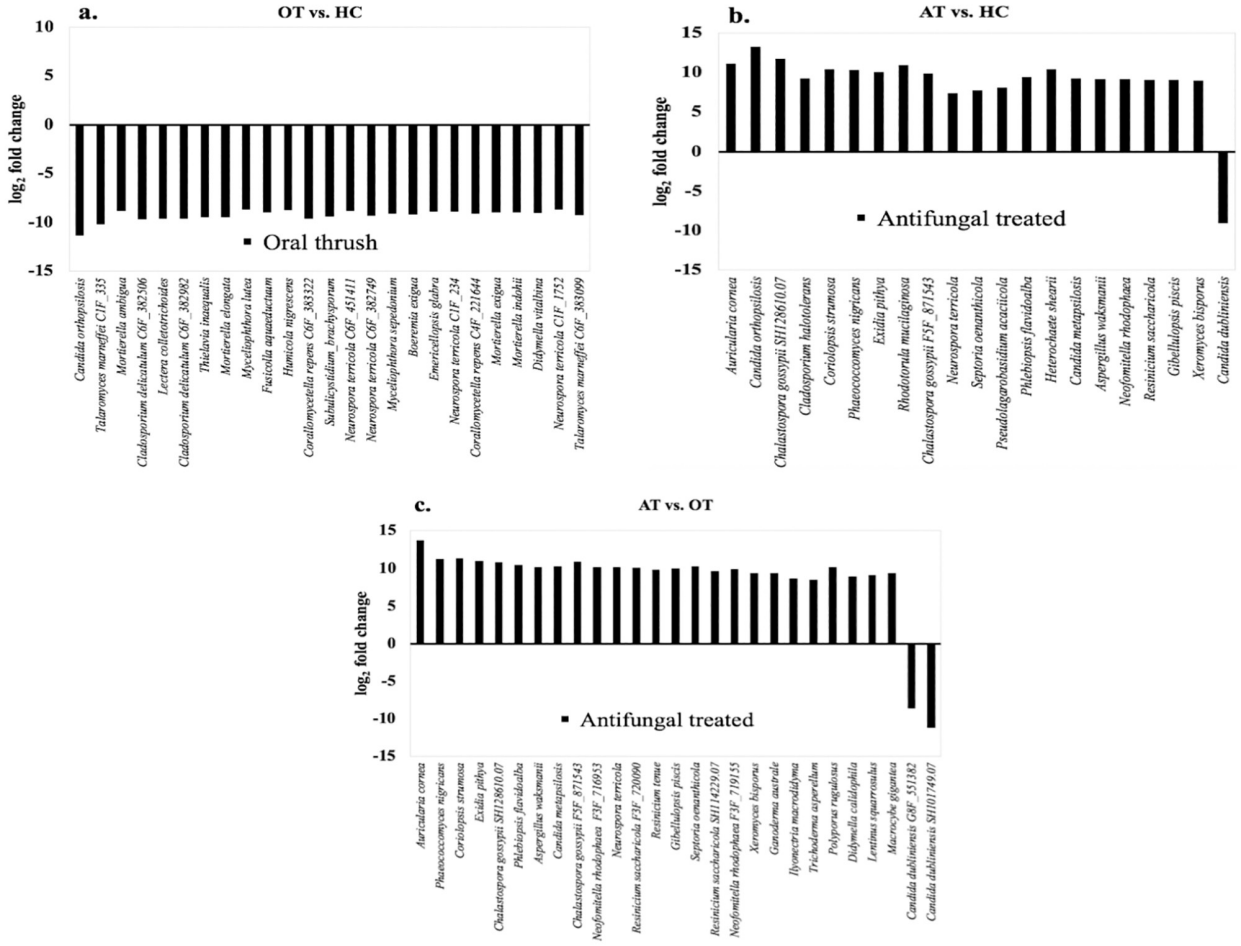
Significant differential abundance (p < 0.05) of fungal taxa within group pairs. a) OT vs. HC (Top 25 strains that showed significant downregulation in OT patients), b) AT vs. HC (A total of 20 strains showed significant upregulation in AT patients, while 1 species showed significant downregulation), c) AT vs. OT (Top 25 strains that showed significant upregulation in AT patients, while only 2 strains showed significant downregulation).

*Differential abundance analysis of fungal taxa between patient group pairs*. Differential abundance analysis was executed to determine statistically significant fungal taxa (log_2_ fold change < -2 or > 2; p < 0.05) based on overabundance or underabundance of fungal species present in the oral samples of OT and AT patients (S7–S9 Tables). Comparison between OT and HC group pairs revealed significant downregulation of 125 fungal taxa in the oral mycoflora of OT patients (S7 Table). The 25 most underabundant fungal species include those in the genera *Candida*, *Talaromyces*, *Cladosporium*, *Lectera*, *Corallomycetella*, *Thielavia*, *Mortierella*, *Subulicystidium*, *Neurospora*, *Phaeosphaeria*, *Malassezia*, *Boeremia*, *Myceliophthora*, *Epicoccum*, *Didymella*, *Trichosporon*, *Arthrobotrys*, *Fusicolla*, and *Guehomyces* (Fig 5a; S7 Table). Overly abundant fungal taxa with a statistical significance of p < 0.05 were not identified in OT patients.

In regard to AT vs. HC study groups, a total of 20 taxa displayed significantly increased abundance in AT patients, while 1 taxon displayed significantly reduced abundance (Fig 5b; S8 Table). Upregulated taxa included *Auricularia*, *Candida*, *Chalastospora*, *Cladosporium*, *Coriolopsis*, *Phaeococcomyces*, *Exidia*, *Rhodotorula*, *Neurospora*, *Septoria*, *Pseudolagarobasidium*, *Phlebiopsis*, *Heterochaete*, *Aspergillus*, *Neofomitella*, *Resinicium*, *Gibellulopsis*, and *Xeromyces*, while downregulation was encountered in *Candida dubliniensis*.

Results from the AT vs. OT group pair highlighted a total of 224 taxa, which had significant differential abundance (S9 Table). The top 25 taxa with increased abundance in AT patients (Fig 5c; S9 Table) were from *Auricularia*, *Phaeococcomyces*, *Coriolopsis*, *Exidia*, *Chalastospora*, *Phlebiopsis*, *Aspergillus*, *Candida*, *Neofomitella*, *Neurospora*, *Resinicium*, *Gibellulopsis*, *Septoria*, *Xeromyces*, *Ganoderma*, *Ilyonectria*, *Trichoderma*, *Polyporus*, *Didymella*, *Lentinus*, and *Macrocybe*, while 2 types of *Candida dubliniensis* strains had decreased proportions.

## Discussion

The increasing isolation of a variety of *Candida* species in oral candidiasis patients poses a great concern to the healthcare professionals [7, 32]. This study showed a significant increase in the frequency of oral *Candida* isolation from OT (68.4%) and AT (61.9%) patients as compared to HC (26.8%). Interestingly, a drop in the percentage of *Candida* isolation from AT group (61.9%) was observed, as compared to the OT group, however, the difference was not significant (p = 1.00). Among the oral *Candida* species isolated via culture, *C*. *albicans* was the most frequently reported, with a significantly higher isolation rate from OT (60.5%, 23 of 38 patients), and AT (42.9%, 9 out of 21 patients), as compared with HC individuals (9.8%, 4 out of 41 patients) (Table 2). The oral *C*. *albicans* isolation rate of the Malaysian patients is almost similar with preceding studies in the Asian regions, including China (75.37%), Iran (64%), and Thailand (61.6%), which exhibited similar predominance of *C*. *albicans*, albeit at a higher incidence in oral candidiasis patients [6, 7, 33].

Moreover, the predominance of *C*. *albicans* was also observed in the oral samples of AT patients (Table 2), thus, inferring the persistence of *C*. *albicans* in the oral cavity of oral thrush patients even after antifungal administration. The finding was also confirmed with oral mycobiota analysis via ITS1 amplicon sequencing, whereby increased AR of *Candida* (Table 4), essentially due to elevated AR of *C*. *albicans*, was noted in AT patients (S2 Table). Hence, relapse of oral thrush infection was highly possible, as AT patients still harboured *C*. *albicans* as the predominant fungal commensal organism (Fig 4). Correspondingly, the recurrence of oral thrush could potentially be associated with antifungal resistance, non-compliant patients, lack of hygiene maintenance towards dental prostheses or having an underlying condition that increases the risk of superficial oral infection as a result of cell-mediated immunodeficiency [3, 34].

On another note, almost one third of the oral *Candida* spp. isolated from OT and AT patients was NAC, while a lower percentage (17.1%) of NAC was isolated from the HC group. However, the proportion of NAC was higher in the HC group (17.1%) as compared to *C*. *albicans* (9.8%) (Table 2). The predominance of NAC in the oral cavity of healthy individuals, as confirmed by the culture method in this study, has not been reported before in the literature, however, a shift towards NAC species associated with oral thrush has been noted [7].

Accordingly, the culture findings in this study showed similar diversity of *Candida* species in OT (n = 7 species) and AT (n = 5 species) patients, as also reported by investigators from other geographical regions, which identified 4–8 *Candida* species [6, 7, 32]. These data reinforce a shift towards co-infection and/or colonization of multiple *Candida* species. In particular, coexistence of different *Candida* species, specifically *C*. *albicans* with NAC species were observed in the oral samples of 10 (26.3%) OT and 2 (9.5%) AT patients, while 3 (7.3%) HC participants had mixed cultures of only NAC species (Table 3). In fact, former and recent cross-sectional studies had consistent pairings of *Candida* species from oral candidiasis infected clinical samples [32, 35–37]. From a medical perspective, this phenomenon needs to be managed effectively as sensitivity to antimycotics vary between *Candida* species [38].

Several studies have cautioned the emergence of NAC species, such as *C*. *glabrata*, which is alerting, as it was cultured from the oral samples of 15.8% of OT patients in this study (Table 2). Studies have focused on reducing the colonization proportions or eradicating *C*. *glabrata*, due to its elevated azole MIC profiles [6], greater presence in adults [39] and candidiasis cases (14%), as confirmed by a European Confederation of Medical Mycology survey [40]. Phylogenetically related species of *C*. *glabrata*, such as *C*. *nivariensis* has been reported in low frequency from oropharyngeal candidiasis [41], which is also in alignment with its rare isolation from oral samples in this study (n = 1/100; Table 2). Sikora et al. [42] advised that this cryptic species may have similar pathogenic mechanisms as *C*. *glabrata* sensu stricto and cautions its multidrug resistance against azole agents, particularly fluconazole, with susceptibility profiles that are equal or lower than *C*. *glabrata*.

Other NAC species, were also of importance, as *C*. *tropicalis* was the most commonly isolated species from the oral sample cultures of OT patients in this cohort (21.1%, n = 8/38, Table 2). Known as a common aetiological agent for invasive candidiasis and bloodstream infections [43], *C*. *tropicalis* can lead to further tissue invasion and greater mortality compared to *C*. *albicans* and other NAC species, as a result of biofilm development, release of proteinases and dimorphic pathogenic mechanisms [44]. To contrast, NAC species such as *C*. *parapsilosis* complex and *C*. *dubliniensis* predominated (14.3%, n = 3/21, Table 2) in the oral sample cultures of AT patients. Typically, individuals with underlying diseases or predisposing factors have high risk of pathogenic dissemination of these species from the oral cavity reservoir into the bloodstream, which may lead to candidaemia [45, 46].

The carrier rate of *Candida* was also affected by local and systemic host factors such as older age, greater risk of malnutrition, denture usage, antibiotic treatment, concurrent bacterial infection, cancer, chemotherapy, hypertension, and xerostomia, which may have played influential roles in the development of oral thrush in this study. Presence of these co-factors were significantly associated with the OT group, as compared to the HC group (Table 1), as also reported by Patel [1] whereby similar associations of predisposing factors have been found with the presence of oral candidiasis or increasing oral *Candida* concentrations.

In this study, the presence of oral thrush manifestations was significantly associated (p = 0.012, Table 1) with OT patients (50%, n = 19), who were elderly (aged 65 years and above), as compared to adults (44.7%, n = 17), and children (5.3%, n = 2; Table 1). A cross-sectional study in Malaysia reported that elderly patients with upper acrylic dentures and removable orthodontic appliances (URA) had a 56.7%– 72.7% oral candidiasis prevalence rate [47]. Significant risk factors for oral candidiasis included older age and denture usage (denture/URA hygiene, overnight wearing of dentures, and daily denture usage of more than 12 hours per day) [47], which resembled the findings of this study. Moreover, old-aged individuals tend to have multiple medical conditions, which affect their oral microecological stability and the health of their oral cavities [48]. Akpan and Morgan [49] discussed that aging patients also had impairment of oral host immune surveillance, which could lead to oral candidiasis. Diminishing salivary flow and higher drug consumption in aging patients were other predispositions for oral candidiasis [6], which may also explain the oral lesions observed in OT patients in this study.

Another significant risk factor for OT patients in this study was denture usage (p = 0.006, Table 1). A total of 63.2% of OT patients with dentures have been significantly correlated with oral thrush in this study (Table 1). Usage and positioning of dentures can form an oral environment with reduced oxygen, low pH, decreased salivary flow to the exposed surfaces. The absence of mechanical tongue cleaning and an anaerobic setting facilitates the development and attachment of *Candida* to acrylic and disrupts the normal host flora [49], which can predispose these individuals to oral candidiasis. Prolonged daily use of dentures have resulted in sore impressions of the oral mucosa and increased *C*. *albicans* load on the contact area of maxillary dentures, leading to oral candidiasis [50]. Overnight wearing of dentures also promotes the growth of *Candida*, as the total amount of saliva produced declines during this time period and elevates the risk of oral candidiasis [51]. Greater *Candida* counts have been linked with hyposalivation [52, 53], with xerostomic patients having greater susceptibility to oral candidiasis [6, 54]. This may explain why a majority of OT patients (61.3%) in this study simultaneously presented with xerostomia (p < 0.001, Table 1).

Hypertension was also a significant comorbidity in 34.2% of OT participants as compared to 7.3% of HC participants in the present study (p = 0.004, Table 1). However, contradicting results have been published in literature. According to recent studies [47, 55, 56], hypertension and hypertensive therapeutics have not been correlated with higher *Candida* colonization rates and oral candidiasis, and that the regular occurrence of hypertension among adults may instead be due to an unhealthy daily routine. In contrast, socioeconomically challenged women in USA [57] and elderly subjects residing in a natural disaster affected town in Japan [58] had significantly increased incidence and colony counts of oral *C*. *albicans* under hypertensive circumstances.

Moreover, the risk of malnutrition was significantly correlated to presence of oral candidiasis in the current study (p = 0.001, Table 1). Malnutrition is a multifactorial issue, whereby BMI was used to reflect the risk of nutritional deficiency in this study, and has been a widely accepted screening tool in clinical settings [59]. OT participants had a 23.7% medium risk and 21.1% high risk of undernutrition, as compared to HC individuals with 4.9% (n = 2) medium risk and 7.3% (n = 3) high risk of malnutrition (Table 1). This finding was however, contradicted with a previous study [60] whereby significant difference of BMI was not identified between oral candidiasis free and oral candidiasis groups.

Systemic bacterial infections were also significantly concurrent with oral thrush in this study (p = 0.004, Table 1). Interkingdom relationships exist in the oral mucosa between bacteria and fungi through symbiosis and antagonism (predation, parasitism, and competition) [61]. Yeasts such as *C*. *albicans* and *C*. *dubliniensis* closely interact with certain oral bacteria, which support the harbouring of co-evolving communities. Hence, development of candidal infections can affect bacterial pathology [62]. Additionally, 21.1% and 13.2% of oral thrush patients had cancer and/or underwent chemotherapy. Theses co-factors were of significance in oral thrush cases (Table 1). Histopathological findings have shown damage to the oral epithelium and mucosal membranes of cancer patients undergoing chemotherapy and/or radiotherapy, in addition to reduced immunological responses [63, 64]. Hence, the host’s reaction to combat *Candida* infection becomes impaired in this scenario and subsequently intensifies the risk of acquiring oral thrush.

The differences in the composition of the oral mycobiota between patients with oral thrush and oral thrush uninfected subjects, and the effect of two weeks of antifungal therapy on the oral mycobiota were demonstrated in this study. In terms of overall diversity, the oral samples of OT patients showed the detection of 144 genera and 187 species, which was lower than that of HC (152 genera, 201 species), while the number of genera and species increased following antifungal treatment (174 genera, 245 species), thereby reflecting the impact of mycobiota diversity on the overgrowth of *Candida* spp. in the oral cavity. The mycobiota alpha diversity was significantly lower in the OT group, as compared to the HC, and AT groups (Fig 1), suggesting that reduced diversity and richness were associated with oral thrush. Diaz et al. [17] also noted lower alpha diversity in cancer patients, whom subsequently manifested oral candidiasis following chemotherapy. This may reinforce the idea that disruption in the diversity of the oral ecosystem can lead to oral diseases, such as oral thrush.

Beta diversity analysis unveiled significant differences in mycobiota composition between OT and HC groups, with noticeable clustering on the PCoA plot (Fig 2a), thus implying the interference of oral candidiasis towards oral homeostatic communities, through disproportionately sustaining the overgrowth of disease-related taxa in the development and progression of this disease [62]. Individuals with immunosuppression (e.g. HIV), oral infections (e.g. dental caries) and/or increased severity of infection also tend to exhibit a varied oral mycobiota profile compared to their healthy counterparts, based on patient group separation on beta diversity PCoA plots [15, 65]. However, Diaz et al. [17] reported that oral candidiasis was not due to fungal compositional imbalance or attainment of a new fungal species, but due to a greater load of certain host mycobiota species (*C*. *albicans* and *C*. *dubliniensis*) in the salivary mycobiota of chemotherapy treated cancer patients.

Using the ITS1 Illumina-Miseq NGS approach, this study confirmed that *C*. *albicans* was the predominant species for oral thrush, with significantly increased AR of *C*. *albicans* in OT vs. HC participants (p = 3.18E-02, Fig 4). These results were typically characteristic for oral thrush patients, as observed in Diaz et al. [17] and Imabayashi et al. [14]. Interestingly, relative abundances of the microbiota and mycobiota in the pulmonary region have been used to differentiate between fungal asymptomatic colonization and symptomatic infection, as well as the severity [66, 67], which was also reflected in this study.

This study also identified *C*. *dubliniensis* as the second most relative abundant species in OT patients via NGS, though it was not significantly prevalent or relative abundant in OT vs. HC or AT vs. OT groups (Fig 4). Previous culture and molecular diagnostic methods support *C*. *albicans* as a major aetiological agent for oral candidiasis, however *C*. *tropicalis* and *C*. *glabrata* are the common NAC species coupled with this oral infection [1, 68, 69]. The incidence and proportion of *C*. *dubliniensis* may have been underestimated in culture, biochemical and molecular investigations due to its indistinguishable morphological appearance to *C*. *albicans*, leading to misidentification and its unknown true correlation with oral candidiasis [70]. In recent ITS1 NGS studies, the relative abundance of *C*. *dubliniensis* in pseudomembranous candidiasis patients was relatively higher than other non-*albicans Candida* spp. [14], suggesting that *C*. *dubliniensis* could play a role in the pathophysiology of oral candidiasis. The pairing of these species were also reinforced by Diaz et al. [17], who reported elevated abundance levels of *C*. *albicans* and *C*. *dubliniensis* in chemotherapy-treated patients with oral candidiasis through NGS of the ITS1 region.

Reports of *in-vivo* fluconazole resistance in *C*. *dubliniensis* strains are limited, but have been observed *in-vitro* [71], which has raised clinical awareness for patients with extended antifungal treatment. This study identified significant downregulation of two *C*. *dubliniensis* strains in AT patients (p = 8.00E-04; p = 5.00E-03) when compared to OT patients (Fig 5). *C*. *dubliniensis*, which was initially part of the core mycobiota in OT patients, had decreased AR following antifungal treatment (AT vs. OT, p = 3.72E-01, Fig 4). As the majority of follow-up patients were treated with nystatin (n = 6/7), this fortifies the sustainable efficacy of this fungicidal drug in the reduction of *C*. *dubliniensis* abundance associated with oral thrush.

However, *Candida* spp. still predominated in all groups (OT, HC, and AT) in terms of PR (100%, Table 4), highlighting the preservation of the oral mycobiota composition, despite presence of oral thrush or antifungal treatment. Essentially, the sustainability of a *Candida*-dominated baseline community among the three study groups in this study was in alignment with other oral thrush studies [14, 17]. Almost similar NGS findings were observed for oral samples of OT and AT patients in this study (Fig 4), despite the fact that AT patients were free of the signs and symptoms of oral thrush upon returning for follow-ups. This suggests that the overgrowth of *Candida* in the oral cavity might not be the sole factor contributing to the clinical presentation of oral thrush. Interestingly, the relative abundance of *C*. *dubliniensis* was lower in the oral samples of AT patients as compared to OT (p = 3.72E-01), and HC (p = 2.82E-02, significant), as observed by the NGS method, suggesting that its growth was suppressed during the recovery process of oral thrush. The relative abundance of “other mycoflora” of AT patients was similar to that of HC, while it was lower in the OT patients, suggesting protective roles of “other mycoflora” against oral thrush (Fig 4; S2 Table). Exploration of other factors, for instance, mycobiota diversity, host defense and microbial proteins, and metabolites, may shed light on the actual pathogenic processes during oral thrush.

Focusing on the core oral mycobiota, it was first defined in a NGS study, as fungal species detected with at least 20% frequency and 1% proportion in healthy individuals [19]. In that study, four common opportunistic genera, *Candida* (75%), *Aspergillus* (35%), *Fusarium* (30%), and *Cryptococcus* (20%) were identified. Among the core mycobiota investigated in this study, *Malassezia* was detected from all oral samples of HC and AT groups, and from 93.75% of oral samples in the OT group (Table 4). *Malassezia* spp. are common colonizers of the oral cavity and have been proposed to be part of the basal oral mycobiota that are crucial in the regulation of oral homeostasis [72]. The identification of a *Malassezia*-dominated community, with low *Candida* loads, amongst patients undergoing chemotherapy without oral candidiasis, has been correlated with deterrence of oral infection [17]. Higher prevalence and proportion of *Malassezia* spp. may hinder the growth of *Streptococcus mutans* and assist in the modulation of oral health against dental caries [13].

Interestingly, a number of fungal species not known to have any prior association with oral infection were identified in this study. Amongst which, the PR of *Neurospora* sp. was significantly higher in HC and AT patients in comparison to OT patients (S3 Table). *Neurospora* has been reported to produce metabolic compounds which are cytotoxic against tumour cell lines—HeLa and MCF-7 [73]. *Myceliophthora lutea*, significantly underabundant in OT vs. HC participants in this study (p = 2.13E-05, Fig 5), has been reported to generate bioactive metabolites, such as isoacremine D, which displayed antimicrobial efficacy against *Staphylococcus aureus* [74]. The phenolics and terpenoids of *Schizophyllum commune* (basidiomycota) were able to induce antidiabetic effects, improve renal function and levels of hepatic enzymes, as well as refining the distribution and proportion of lipid and antioxidants, in streptozotocin-induced diabetic Wistar rats [75]. *Trichoderma asperellum* expressed biocontrol properties against *Fusarium xylarioides* (associated with coffee wild disease), *Magnaporthiopsis maydis* (maize late wilt disease), *Fusarium graminearum*, and *Fusarium oxysporum* (inoculated onto roots of cucumber seedlings) through enzyme-related mycoparasitism, the release of antimicrobial peptides and primary and secondary metabolites with fungicidal effects [76–78]. These species, thought to be acquired from the environment, may be beneficial in the regulation of a healthy oral mycobiota, however further research into the metabolite profile, mechanism of mycoparasitism, molecular systems, and functional applications of these fungi are necessary to validate the findings.

Different laboratory techniques have varying degrees of diagnostic capability, which can reveal important characteristics of an infection to reach a clinical diagnosis [79]. In the current study, Gram stains posed as an unreliable screening method for the laboratory diagnosis of oral thrush as the positive rate was approximately six times lower as compared to the conventional culture method on BCA (78.9%, n = 30, Table 2). Whereas, confirmation of *C*. *albicans* as the main pathological agent in oral thrush was possible through culture, molecular, and NGS applications in this study. However, as *Candida* spp. was not isolated from culture in about 30% of patients clinically diagnosed with oral thrush, this suggests the insensitivity of the cultural method as the yeasts were able to be detected via amplicon sequencing in all 30 samples.

In our observation, culture was only valuable for isolation of fungi when coupled with clinical diagnosis, whereby NGS, as a sole method, behaved as a diagnostic marker, through exhibiting relative abundance values that reflected the pathophysiology of a disease. NGS may also be useful in determining the mycobiota composition for recurrent or recalcitrant oral candidiasis, where regular treatment is ineffective. Some pathogenic species cannot be cultured or may have been missed in routine diagnosis, hence, in these scenarios, evaluating the whole mycobiota profile in a patient can aid in the tailoring of antimycotic therapies better suited for a patient [80].

### Limitations and future perspectives

Oral mycobiota papers reported an average sequence length of 151 bp [81], 248 bp [19], and 200–400 bp [14] for ITS1 subregions. Consequently, the outcome was zero detection of *C*. *glabrata* species [82] via NGS approach in this study, as also reported by Imabayashi et al. [14]. It is concerning as *C*. *glabrata* is typically isolated from oral candidiasis patients using conventional culture methods [32, 37] and has reduced antifungal susceptibility patterns to azoles. These results emphasised that culture-based studies are essential in species typing and antifungal testing for more effective treatment of oral thrush and surveillance of antifungal resistance. Other species such as *C*. *nivariensis* and *Saccharomyces cerevisiae*, which were cultured on BCA in this study, were also not discovered through ITS1 amplicon sequencing. This could be due to the inability to delineate these species based on the Illumina Miseq ITS1 amplicon sequence length (250 bp). Fungal species which have longer ITS1 regions (for instance, *C*. *glabrata*– 520 bp; *C*. *nivariensis*– 361bp; *S*. *cerevisiae*– 460 bp) require more complete sequences for exact species identification [82, 83].

Unfortunately, there is a lack of alternative pan-fungal regions that provide sufficient variability for the delineation of fungal taxonomies at the phylum, class, order, family, genus and species level [80], thus reliance on the ITS region is currently inevitable. Certain DNA sequences at the genus and species level could not be annotated and were labelled as “fungi”, “uncultured fungi” or “unidentified fungi” from the UNITE database, as they may have originated from non-target sequences, mismatched sequences, primer artifacts [72], incomplete sequence archives, or had limited variability for the delineation of taxonomies [84]. Such sequences can perturb diversity calculations [85], and thus were removed from the relative abundance dataset in this study. However, it is imperative to note that unidentified sequences may be potential fungi awaiting discovery with continued research in identifying and depositing more sequences to update fungal nomenclature or with the aid of more refined culture-independent techniques.

Reference databases contain publicly deposited sequences, and have been known to contain sequencing errors, that may lead to misidentification of taxonomies, with a 20% error rate at species level [86]. Moreover, persistence of insufficient intraspecies variability, based on the inability to discriminate ITS sequences of *C*. *albicans* and *C*. *africana* due to a high degree of homology [87], may lead to false diversity estimates.

Exploration into interspecies (co-occurrence of fungal genera) with Pearson correlation using Fast-Spar algorithm [88], and interkingdom relationships between bacteria and fungi using cross-kingdom association networks [89], were not possible for this study due to budgetary constraints, however it may reveal essential interactions that contribute to the exacerbation of oral thrush. The simultaneous occurrences of a wide variety of oral organisms have supported an ecological balance through physical, chemical and metabolic exchanges, that can trigger or circumvent oral diseases [81]. Diaz et al. [17] proposed that a lower diversity and higher abundance of aciduric bacteria in combination with a *Candida* mycotype, may predispose chemotherapy-induced cancer patients to oropharyngeal candidiasis, as well as heighten the risk of caries [90, 91].

Notably, a small sample size may have resulted in limited confidence and biased associations with host-related factors, thus caution should be exercised during interpretation of these findings. In view of the current results, a larger sample size may be warranted for correlation of patient-related factors with oral thrush for future studies. Longitudinal study and testing of a larger sample size should also be implemented for the improved evaluation of oral mycobiota features in oral thrush participants.

## Conclusion

The different diagnostic approaches (culture, molecular, and ITS1 amplicon sequencing) applied in this study could detect the predominant oral fungal pathogen, *Candida* spp., across all participant groups. The lack of ability to discriminate between presence and absence of oral thrush infection via the culture methods, alludes to the fact that it is only reliable in conjunction with clinical diagnosis. However, ITS1 amplicon analysis further exhibited differences in phylogenetic diversity and taxonomical abundance. NGS relayed the significant increase in relative abundance of *C*. *albicans* in OT vs. HC participants, alongside conveying the differences in species richness, diversity (alpha diversity), and oral mycobiota composition (beta diversity) with regards to oral thrush status, thus providing a more wholistic understanding on the pathophysiology of oral thrush, which can be targeted to improve oral homeostasis.

## Supporting information

S1 Table Demographical information of OT, HC and AT participants for ITS1 amplicon analysis. (DOCX)

S2 Table Relative abundance of oral fungi at species level (top 10 most abundant in each study group; arranged from highest to lowest species relative abundance in OT patients). (DOCX)

S3 Table Prevalence of oral fungi at species level (top 10 most abundant in each study group; arranged from highest to lowest species relative abundance in OT patients). (DOCX)

S4 Table Prevalence and relative abundance of significant oral fungal species in OT vs. HC groups (arranged from most to least significant relative abundance). (DOCX)

S5 Table Prevalence and relative abundance of significant oral fungal species in AT vs. OT groups (arranged from most to least significant relative abundance). (DOCX)

S6 Table Prevalence and relative abundance of significant oral fungal species in AT vs. HC groups (arranged from most to least significant relative abundance). (DOCX)

S7 Table Significant underabundance of the top 25 strains in OT vs. HC oral rinse samples. (DOCX)

S8 Table Significant differential abundance (20 overabundant and 1 underabundant) of fungal strains in AT vs. HC oral rinse samples. (DOCX)

S9 Table Significant differential abundance (top 25 overabundant, 2 underabundant) of fungal strains in AT vs. OT oral rinse samples. (DOCX)

S10 Table Alpha diversity index values for OT, HC and AT groups. (DOCX)

S1 File Chao-1 bias-corrected and Shannon alpha diversity index raw values. (XLSX)

S2 File Bray-Curtis, Jaccard, Euclidean, Unweighted UniFrac, Weighted UniFrac beta diversity raw values for OT, HC and AT groups. (XLSX)
