# Supplementary material for: Candida species and oral mycobiota of patients clinically diagnosed with oral thrush
Source: PLoS One. 2023 Apr 17;18(4):e0284043. doi: 10.1371/journal.pone.0284043 (PMC10109505; doi:10.1371/journal.pone.0284043)
Supplement: S1 Table — (DOCX) [file pone.0284043.s001.docx]

**S1 Table. Demographical information of OT, HC and AT participants for ITS1 amplicon analysis.**

| **Participant demographics** | **No. of participants, n=30 (%)** | | |
| --- | --- | --- | --- |
|  | **Oral thrush (OT)**  **n=16 (%)** | **Healthy control (HC)**  **n=7 (%)** | **Follow-up^a^ (AT)**  **n=7 (%)** |
| **Gender (Female)** | 23 (60.5) | 20 (48.8) | 13 (61.2) |
| **Age group (Elderly)** | 11 (69) | 3 (43) | 5 (71) |
| **Age mean ± SD (years)** | 66.6 ± 17.7 | 55.9 ± 22.1 | 62.7 ± 20.3 |
| **Age range (years)** | 18 – 83 | 22 – 78 | 18 - 77 |
| **Ethnicity (Chinese)** | 9 (56) | 4 (57) | 3 (43) |
| **Smoker (Yes)** | 1 (6) | 3 (43) | 0 (0) |
| **Risk of malnutrition^b^** | | | |
| **Low** | 8 (50) | 4 (57) | 6 (86) |
| **Medium** | 2 (13) | 1 (14) | 0 (0) |
| **High** | 4 (25) | 1 (14) | 1 (14) |
| **Antimicrobial wash (Yes)** | 2 (12.5) | 2 (29) | 0 (0) |
| **Denture usage (Yes)^c^** | 5 (31) | 2 (29) | 2 (29) |
| **Topical/inhalational corticosteroid (Yes)** | 4 (25) | 1 (14) | 3 (43) |
| **Antibiotic treatment^d^** | 5 (31) | 0 (0) | 0 (0) |
| **Concurrent bacterial infection (Yes)^e^** | 4 (25) | 0 (0) | 0 (0) |
| **Cancer (Yes)^f^** | 5 (31) | 0 (0) | 1 (14) |
| **Chemotherapy (Yes)** | 4 (25) | 0 (0) | 0 (0) |
| **Type 2 diabetes (Yes)** | 3 (19) | 2 (29) | 1 (14) |
| **Dyslipidaemia (Yes)** | 5 (31) | 2 (29) | 4 (57) |
| **HIV (Yes)** | 1 (6) | 0 (0) | 0 (0) |
| **Hypertension (Yes)** | 7 (44) | 1 (14) | 5 (71) |
| **Xerostomia (Yes)** | 7 (44) | 0 (0) | 0 (0) |
| **Type of candidiasis** | | | |
| **Pseudomembranous** | 10 (62.5) | 0 (0) | 0 (0) |
| **Erythematous** | 2 (12.5) | 0 (0) | 0 (0) |
| **Angular cheilitis** | 1 (6.3) | 0 (0) | 0 (0) |
| **Median rhomboid glossitis** | 2 (12.5) | 0 (0) | 0 (0) |
| **Denture stomatitis** | 2 (12.5) | 0 (0) | 0 (0) |

^a^Patient-related factors for AT patients (subgroup of OT patients) were not included in beta diversity analysis to prevent double-counting

^b^Three patients preferred not to disclose information related to weight, height and certain clinical conditions

^c^Two OT and AT patients had partial dentures: Upper denture (n=1) and lower denture (n=1); One HC participant had partial dentures (upper)

^d^Antibiotic treatment prescribed to oral thrush patients included Anti-Tuberculosis drug (n=1), Piperacillin/tazobactam (n=2), Augmentin (n=1) and Meropenem and Piperacillin/tazobactam (n=1)

^e^Types of disseminated concurrent bacterial infections: Two respiratory (1 *Mycobacterium tuberculosis* and 1 undefined bacteria), One bloodstream (1 undefined Gram negative bacteria), and One bloodstream and genitourinary (*Escherichia coli* and *Klebsiella*)

^f^Solid organ cancer in OT and AT patients
