# Supplementary material for: Candida species and oral mycobiota of patients clinically diagnosed with oral thrush
Source: PLoS One. 2023 Apr 17;18(4):e0284043. doi: 10.1371/journal.pone.0284043 (PMC10109505; doi:10.1371/journal.pone.0284043)
Supplement: S2 Table — (DOCX) [file pone.0284043.s002.docx]

**S2 Table. Relative abundance of oral fungi at species level (top 10 most abundant in each study group; arranged from highest to lowest species relative abundance in OT patients).**

| **Species**  **(n=19)** | **Relative Abundance** | | | **p-value** | | |
| --- | --- | --- | --- | --- | --- | --- |
|  | **Oral thrush (OT)** | **Healthy control (HC)** | **Follow-up (AT)** | **OT vs. HC** | **AT vs. OT** | **AT vs. HC** |
| ***Candida albicans*** | 7.27E-01 | 5.41E-01 | 7.93E-01 | 3.18E-02* | 6.67E-01 | 1.89E-01 |
| ***Candida dubliniensis*** | 2.02E-01 | 1.22E-01 | 3.69E-05 | 8.97E-01 | 1.70E-01 | 2.82E-02* |
| ***Aspergillus penicillioides*** | 2.24E-02 | 4.18E-03 | 2.86E-03 | 5.23E-01 | 5.34E-01 | 8.25E-01 |
| ***Candida tropicalis*** | 1.11E-02 | 1.49E-03 | 7.18E-04 | 2.71E-01 | 1.72E-01 | 2.49E-01 |
| ***Candida parapsilosis*** | 6.97E-03 | 4.15E-03 | 1.10E-03 | 7.37E-01 | 4.40E-01 | 1.86E-01 |
| ***Gliocladium cibotii*** | 6.43E-03 | 0 | 0 | 5.21E-01 | 5.21E-01 | 0 |
| ***Malassezia restricta*** | 2.64E-03 | 9.63E-03 | 9.00E-03 | 2.45E-01 | 2.02E-01 | 9.15E-01 |
| ***Byssochlamys lagunculariae*** | 2.14E-03 | 2.10E-03 | 1.07E-02 | 1.94E-01 | 2.08E-01 | 6.27E-01 |
| ***Neurospora terricola*** | 1.41E-03 | 4.90E-02 | 1.29E-02 | 7.07E-03* | 1.42E-01 | 1.90E-01 |
| ***Schizophyllum commune*** | 1.11E-03 | 7.66E-04 | 4.74E-03 | 5.99E-01 | 2.52E-01 | 4.53E-02* |
| ***Malassezia globosa*** | 6.39E-04 | 2.15E-03 | 6.79E-03 | 3.62E-01 | 1.50E-01 | 4.13E-01 |
| ***Gibellulopsis nigrescens*** | 5.13E-04 | 1.34E-02 | 4.82E-03 | 6.51E-03* | 5.39E-02 | 2.25E-01 |
| ***Corallomycetella repens*** | 3.34E-04 | 1.17E-02 | 1.87E-04 | 1.27E-01 | 7.25E-01 | 3.18E-01 |
| ***Myceliophthora lutea*** | 2.74E-04 | 1.38E-02 | 3.32E-03 | 5.42E-03* | 1.40E-01 | 1.78E-01 |
| ***Trichosporon asahii*** | 1.16E-04 | 5.40E-02 | 6.87E-04 | 1.34E-01 | 7.62E-02 | 3.43E-01 |
| ***Trichoderma asperellum*** | 1.16E-04 | 1.70E-02 | 5.35E-03 | 1.36E-01 | 3.70E-02* | 5.10E-01 |
| ***Sampaiozyma vanillica*** | 1.05E-04 | 1.52E-02 | 2.62E-02 | 1.29E-01 | 1.29E-01 | 4.91E-01 |
| ***Chalastospora gossypii*** | 2.57E-05 | 3.72E-06 | 5.25E-03 | 3.54E-01 | 3.33E-02* | 1.68E-01 |
| ***Staphylotrichum coccosporum*** | 2.18E-05 | 4.46E-05 | 6.89E-03 | 6.56E-01 | 1.33E-01 | 3.38E-01 |
| **Others** | 1.51E-02 | 1.19E-02 | 1.05E-01 | - | - | - |

*p < 0.05, statistically significant, independent student t-test
