# Supplementary material for: Candida species and oral mycobiota of patients clinically diagnosed with oral thrush
Source: PLoS One. 2023 Apr 17;18(4):e0284043. doi: 10.1371/journal.pone.0284043 (PMC10109505; doi:10.1371/journal.pone.0284043)
Supplement: S3 Table — (DOCX) [file pone.0284043.s003.docx]

**S3 Table. Prevalence of oral fungi at species level (top 10 most abundant in each study group; arranged from highest to lowest species relative abundance in OT patients).**

| **Species**  **(n=19)** | **Prevalence, n (%)** | | | **p-value** | | |
| --- | --- | --- | --- | --- | --- | --- |
|  | **Oral thrush (OT)**  **n=16 (%)** | **Healthy Control (HC)**  **n=7 (%)** | **Follow-up (AT)**  **n=7 (%)** | **OT vs. HC** | **AT vs. OT** | **AT vs. HC** |
| ***Candida albicans*** | 16  (100) | 7  (100) | 7  (100) | - | - | - |
| ***Candida dubliniensis*** | 14  (87.5) | 5  (71.43) | 5  (71.43) | 3.72E-01 | 3.72E-01 | 1 |
| ***Aspergillus penicillioides*** | 6  (37.5) | 3  (42.86) | 7  (100) | 8.19E-01 | 3.71E-03* | 1.52E-02* |
| ***Candida tropicalis*** | 13 (81.25) | 4  (57.14) | 3  (42.86) | 2.45E-01 | 7.05E-02 | 6.26E-01 |
| ***Candida parapsilosis*** | 7  (43.75) | 5  (71.43) | 4  (57.14) | 2.40E-01 | 5.75E-01 | 6.11E-01 |
| ***Gliocladium cibotii*** | 3  (18.75) | 0  (0) | 0  (0) | 2.38E-01 | 2.38E-01 | - |
| ***Malassezia restricta*** | 15 (93.75) | 7  (100) | 7  (100) | 5.21E-01 | 5.21E-01 | - |
| ***Byssochlamys lagunculariae*** | 1  (6.25) | 1  (14.29) | 2  (28.57) | 5.51E-01 | 1.57E-01 | 5.52E-01 |
| ***Neurospora terricola*** | 5  (31.25) | 7  (100) | 7  (100) | 1.18E-03** | 1.18E-03** | - |
| ***Schizophyllum commune*** | 6  (37.5) | 4  (57.14) | 6  (85.71) | 4.05E-01 | 3.38E-02* | 2.71E-01 |
| ***Malassezia globosa*** | 7  (43.75) | 6  (85.71) | 6  (85.71) | 6.62E-02 | 6.62E-02 | 1 |
| ***Gibellulopsis nigrescens*** | 5  (31.25) | 4  (57.14) | 6  (85.71) | 2.62E-01 | 1.47E-02* | 2.71E-01 |
| ***Corallomycetella repens*** | 1  (6.25) | 1  (14.29) | 2  (28.57) | 1.57E-01 | 4.72E-03** | 3.17E-01 |
| ***Myceliophthora lutea*** | 4  (25) | 6  (85.71) | 4  (57.14) | 5.11E-03* | 1.49E-01 | 2.71E-01 |
| ***Trichosporon asahii*** | 6  (37.5) | 3  (42.86) | 6  (85.71) | 8.19E-01 | 3.38E-02* | 1.09E-01 |
| ***Trichoderma asperellum*** | 2  (12.5) | 1  (14.29) | 5  (71.43) | 9.12E-01 | 3.09E-03** | 3.06E-02* |
| ***Sampaiozyma vanillica*** | 2  (12.5) | 2  (28.57) | 5  (71.43) | 3.72E-01 | 3.09E-03* | 1.26E-01 |
| ***Chalastospora gossypii*** | 5  (31.25) | 1  (14.29) | 3  (42.86) | 4.17E-01 | 6.10E-01 | 2.71E-01 |
| ***Staphylotrichum coccosporum*** | 1  (6.25) | 1  (14.29) | 2  (28.57) | 5.51E-01 | 1.57E-01 | 5.52E-01 |
| **Others** | 16  (100) | 7  (100) | 7  (100) | - | - | - |

*p < 0.05, statistically significant, independent student t-test

**p < 0.005, very statistically significant, independent student t-test
