# Supplementary material for: Candida species and oral mycobiota of patients clinically diagnosed with oral thrush
Source: PLoS One. 2023 Apr 17;18(4):e0284043. doi: 10.1371/journal.pone.0284043 (PMC10109505; doi:10.1371/journal.pone.0284043)
Supplement: S4 Table — (DOCX) [file pone.0284043.s004.docx]

**S4 Table. Prevalence and relative abundance of significant oral fungal species in OT vs. HC groups (arranged from most to least significant relative abundance).**

| Species  (n=38) | Prevalence, n (%) | | Relative Abundance | | Relative abundance p-value |
| --- | --- | --- | --- | --- | --- |
|  | **Oral thrush (OT)**  **n=16 (%)** | **Healthy control (HC)**  **n=7 (%)** | **Oral thrush (OT)** | **Healthy control (HC)** |  |
| ***Penicillium pimiteouiense*** | 1 (6.25) | 4 (57.1) | 2.77E-05 | 1.63E-03 | 5.33E-04 |
| ***Fusarium oxysporum*** | 12 (75) | 7 (100) | 3.60E-04 | 5.39E-03 | 9.40E-04 |
| ***Chaetomium brasiliense*** | 3 (18.75) | 4 (57.1) | 9.89E-05 | 8.15E-03 | 1.17E-03 |
| ***Gymnoascus reessii*** | 1 (6.25) | 4 (57.1) | 6.23E-05 | 2.55E-03 | 2.44E-03 |
| ***Mortierella indohii*** | 4 (25) | 4 (57.1) | 2.23E-04 | 9.87E-03 | 3.14 E-03 |
| ***Mortierella gamsii*** | 2 (12.5) | 3 (42.9) | 2.97E-06 | 4.69E-04 | 3.55E-03 |
| ***Cystofilobasidium infirmominiatum*** | 2 (12.5) | 3 (42.9) | 1.98E-05 | 9.78E-04 | 3.78E-03 |
| ***Talaromyces marneffei*** | 0 (0) | 5 (71.4) | 0 (0) | 2.71E-03 | 4.36E-03 |
| ***Hamigera pallida*** | 3 (18.75) | 3 (42.9) | 9.89E-06 | 1.32E-03 | 5.18E-03 |
| ***Myceliophthora lutea*** | 4 (25) | 6 (85.7) | 2.74E-04 | 1.38E-02 | 5.42E-03 |
| ***Mortierella elongata*** | 4 (25) | 4 (57.1) | 5.88E-04 | 6.40E-03 | 5.95E-03 |
| ***Fusicolla aquaeductuum*** | 1 (6.25) | 4 (57.1) | 9.89E-07 | 1.08E-03 | 6.31E-03 |
| ***Mortierella alpina*** | 10 (62.5) | 5 (71.4) | 1.25E-04 | 2.88E-03 | 6.51E-03 |
| ***Gibellulopsis nigrescens*** | 5 (31.25) | 4 (57.1) | 5.13E-04 | 1.34E-02 | 6.51E-03 |
| ***Neurospora terricola*** | 5 (31.25) | 7 (100) | 1.41E-03 | 4.90E-02 | 7.07E-03 |
| ***Guehomyces pullulans*** | 3 (18.75) | 3 (42.9) | 3.56E-05 | 1.32E-03 | 1.06E-02 |
| ***Mortierella ambigua*** | 0 (0) | 3 (42.9) | 0 (0) | 3.60E-04 | 1.55E-02 |
| ***Arthrobotrys thaumasia*** | 1 (6.25) | 3 (42.9) | 5.93E-06 | 2.19E-03 | 1.90E-02 |
| ***Wardomycopsis inopinata*** | 1 (6.25) | 3 (42.9) | 1.98E-06 | 8.96E-04 | 1.93E-02 |
| ***Scedosporium dehoogii*** | 0 (0) | 2 (28.6) | 0 (0) | 7.18E-04 | 2.00E-02 |
| ***Pseudallescheria angusta*** | 1 (6.25) | 2 (28.6) | 4.94E-06 | 9.45E-04 | 2.03E-02 |
| ***Myceliophthora sepedonium*** | 2 (12.5) | 5 (71.4) | 3.66E-05 | 1.82E-03 | 2.17E-02 |
| *Humicola nigrescens* | 0 (0) | 3 (42.9) | 0 (0) | 6.32E-04 | 2.35E-02 |
| *Corynascella humicola* | 1 (6.25) | 3 (42.9) | 4.94E-06 | 8.00E-04 | 2.39E-02 |
| *Emericellopsis humicola* | 0 (0) | 2 (28.6) | 0 (0) | 1.41E-04 | 2.49E-02 |
| *Arthrographis kalrae* | 0 (0) | 2 (28.6) | 0 (0) | 7.74E-04 | 2.50E-02 |
| *Emericellopsis glabra* | 0 (0) | 2 (28.6) | 0 (0) | 7.18E-04 | 2.51E-02 |
| *Penicillium oxalicum* | 0 (0) | 2 (28.6) | 0 (0) | 4.76E-04 | 2.57E-02 |
| *Thermomyces lanuginosus* | 2 (12.5) | 3 (42.9) | 2.77E-05 | 6.14E-04 | 2.60E-02 |
| *Kazachstania humilis* | 0 (0) | 2 (28.6) | 0 (0) | 3.24E-04 | 2.62E-02 |
| *Penicillium astrolabium* | 0 (0) | 2 (28.6) | 0 (0) | 1.41E-04 | 2.67E-02 |
| *Fuscoporia ferrea* | 0 (0) | 2 (28.6) | 0 (0) | 2.75E-04 | 2.69E-02 |
| *Exophiala dermatitidis* | 2 (12.5) | 2 (28.6) | 2.97E-05 | 8.03E-04 | 2.77E-02 |
| *Aspergillus sydowii* | 0 (0) | 2 (28.6) | 0 (0) | 2.60E-05 | 2.95E-02 |
| *Chaetomium grande* | 6 (37.5) | 3 (42.9) | 7.02E-04 | 2.58E-03 | 3.09E-02 |
| *Candida albicans* | 7 (43.75) | 7 (100) | 7.27E-01 | 5.41E-01 | 3.18E-02 |
| *Microporus affinis* | 1 (6.25) | 2 (28.6) | 1.98E-06 | 1.75E-04 | 3.23E-02 |
| *Aspergillus amstelodami* | 1 (6.25) | 3 (42.9) | 9.89E-07 | 1.16E-03 | 4.28E-02 |
