# Supplementary material for: Candida species and oral mycobiota of patients clinically diagnosed with oral thrush
Source: PLoS One. 2023 Apr 17;18(4):e0284043. doi: 10.1371/journal.pone.0284043 (PMC10109505; doi:10.1371/journal.pone.0284043)
Supplement: S5 Table — (DOCX) [file pone.0284043.s005.docx]

**S5 Table. Prevalence and relative abundance of significant oral fungal species in AT vs. OT groups (arranged from most to least significant relative abundance).**

| **Species**  **(n=17)** | **Prevalence, n (%)** | | **Relative Abundance** | | **Relative abundance p-value** |
| --- | --- | --- | --- | --- | --- |
|  | **Oral thrush (OT)**  **n=16 (%)** | **Follow-up (AT)**  **n=7 (%)** | **Oral thrush (OT)** | **Follow-up (AT)** |  |
| ***Fusarium oxysporum*** | 12 (75) | 7 (100) | 3.60E-04 | 4.06E-03 | 7.70E-03 |
| ***Auricularia cornea*** | 0 (0) | 6 (85.7) | 0 (0) | 2.42E-03 | 1.69E-02 |
| ***Didymella vitalbina*** | 0 (0) | 2 (28.6) | 0 (0) | 1.14E-05 | 2.55E-02 |
| ***Mortierella alpina*** | 10 (62.5) | 5 (71.4) | 1.25E-04 | 1.54E-03 | 3.07E-02 |
| ***Didymella calidophila*** | 1 (6.25) | 3 (42.9) | 1.98E-06 | 1.49E-05 | 3.08E-02 |
| ***Lentinus squarrosulus*** | 0 (0) | 3 (42.9) | 0 (0) | 1.42E-04 | 3.12E-02 |
| ***Aspergillus thermomutatus*** | 0 (0) | 2 (28.6) | 0 (0) | 2.84E-05 | 3.29E-02 |
| ***Chalastospora gossypii*** | 5 (31.25) | 3 (42.9) | 2.57E-05 | 3.72E-06 | 3.33E-02 |
| ***Malassezia furfur*** | 0 (0) | 3 (42.9) | 0 (0) | 4.82E-05 | 3.57E-02 |
| ***Trichoderma asperellum*** | 2 (12.5) | 5 (71.4) | 1.16E-04 | 5.35E-03 | 3.70E-02 |
| ***Hortaea werneckii*** | 1 (6.25) | 2 (28.6) | 9.89E-07 | 8.44E-04 | 3.77E-02 |
| ***Earliella scabrosa*** | 6 (37.5) | 6 (85.7) | 2.16E-04 | 2.49E-04 | 3.91E-02 |
| ***Grammothele lineata*** | 5 (31.25) | 4 (57.1) | 5.24E-05 | 0 (0) | 4.10E-02 |
| ***Coniosporium apollinis*** | 0 (0) | 2 (28.6) | 0 (0) | 3.41E-05 | 4.11E-02 |
| ***Cladosporium delicatulum*** | 9 (56.25) | 4 (57.1) | 1.38E-04 | 7.28E-03 | 4.49E-02 |
| ***Vishniacozyma victoriae*** | 4 (25) | 4 (57.1) | 3.16E-05 | 6.47E-03 | 4.99E-02 |
| ***Resinicium saccharicola*** | 1 (6.25) | 3 (42.9) | 9.89E-07 | 6.30E-04 | 4.99E-02 |
