# Supplementary material for: Candida species and oral mycobiota of patients clinically diagnosed with oral thrush
Source: PLoS One. 2023 Apr 17;18(4):e0284043. doi: 10.1371/journal.pone.0284043 (PMC10109505; doi:10.1371/journal.pone.0284043)
Supplement: S6 Table — (DOCX) [file pone.0284043.s006.docx]

**S6 Table. Prevalence and relative abundance of significant oral fungal species in AT vs. HC groups (arranged from most to least significant relative abundance).**

| **Species**  **(n=2)** | **Prevalence, n (%)** | | **Relative Abundance** | | **Relative abundance p-value** |
| --- | --- | --- | --- | --- | --- |
|  | **Follow-up (AT)**  **n=16 (%)** | **Healthy control (HC)**  **n=7 (%)** | **Follow-up (AT)** | **Healthy control (HC)** |  |
| ***Candida dubliniensis*** | 5 | 5 | 3.69E-05 | 1.21E-01 | 2.82E-02 |
| ***Schizophyllum commune*** | 6 | 4 | 4.74E-03 | 7.66E-04 | 4.53E-02 |
