# Supplementary material for: Candida species and oral mycobiota of patients clinically diagnosed with oral thrush
Source: PLoS One. 2023 Apr 17;18(4):e0284043. doi: 10.1371/journal.pone.0284043 (PMC10109505; doi:10.1371/journal.pone.0284043)
Supplement: S7 Table — (DOCX) [file pone.0284043.s007.docx]

**S7 Table. Significant underabundance of the top 25 strains in OT vs. HC oral rinse samples.**

| **Species^a^** | **Log_2_ Fold change** | **Fold change** | **p*-*value** | **FDR p*-*value** | **Bonferroni p*-*value** |
| --- | --- | --- | --- | --- | --- |
| ***Candida orthopsilosis*** | -11.33 | -2582.89 | 8.02E-11 | 1.76E-07 | 1.76E-07 |
| ***Talaromyces marneffei* C1F_335** | -10.24 | -1206.68 | 3.29E-10 | 3.62E-07 | 7.23E-07 |
| ***Mortierella ambigua*** | -8.87 | -466.85 | 5.73E-09 | 7.66E-07 | 1.26E-05 |
| ***Cladosporium delicatulum* C6F_382506** | -9.70 | -833.91 | 6.58E-09 | 7.66E-07 | 1.45E-05 |
| ***Lectera colletotrichoides*** | -9.65 | -801.46 | 7.43E-09 | 7.66E-07 | 1.63E-05 |
| ***Cladosporium delicatulum* C6F_382982** | -9.67 | -812.29 | 7.66E-09 | 7.66E-07 | 1.68E-05 |
| ***Thielavia inaequalis*** | -9.50 | -722.77 | 8.81E-09 | 7.66E-07 | 1.94E-05 |
| ***Mortierella elongata*** | -9.50 | -722.77 | 8.97E-09 | 7.66E-07 | 1.97E-05 |
| ***Myceliophthora lutea*** | -8.72 | -420.59 | 9.70E-09 | 7.66E-07 | 2.13E-05 |
| ***Fusicolla aquaeductuum*** | -8.97 | -502.56 | 9.72E-09 | 7.66E-07 | 2.14E-05 |
| ***Humicola nigrescens*** | -8.80 | -447.13 | 1.23E-08 | 7.66E-07 | 2.70E-05 |
| ***Corallomycetella repens* C6F_383322** | -9.63 | -792.92 | 1.35E-08 | 7.66E-07 | 2.97E-05 |
| ***Subulicystidium_brachysporum*** | -9.44 | -692.80 | 1.37E-08 | 7.66E-07 | 3.00E-05 |
| ***Neurospora terricola* C6F_451411** | -8.88 | -469.58 | 1.39E-08 | 7.66E-07 | 3.06E-05 |
| ***Neurospora terricola* C6F_382749** | -9.34 | -648.80 | 1.46E-08 | 7.66E-07 | 3.22E-05 |
| ***Myceliophthora sepedonium*** | -9.13 | -561.176 | 1.58E-08 | 7.66E-07 | 3.46E-05 |
| ***Boeremia exigua*** | -9.18 | -580.50 | 1.82E-08 | 7.66E-07 | 4.01E-05 |
| ***Emericellopsis glabra*** | -8.92 | -484.71 | 1.92E-08 | 7.66E-07 | 4.23E-05 |
| ***Neurospora terricola* C1F_234** | -8.96 | -496.33 | 1.96E-08 | 7.66E-07 | 4.31E-05 |
| ***Corallomycetella repens* C4F_221644** | -9.10 | -549.54 | 2.10E-08 | 7.66E-07 | 4.62E-05 |
| ***Mortierella exigua*** | -9.01 | -517.01 | 2.44E-08 | 7.66E-07 | 5.38E-05 |
| ***Mortierella indohii*** | -9.00 | -511.47 | 2.47E-08 | 7.66E-07 | 5.42E-05 |
| ***Didymella vitalbina*** | -9.06 | -534.91 | 2.55E-08 | 7.66E-07 | 5.60E-05 |
| ***Neurospora terricola* C1F_1752** | -8.68 | -410.86 | 2.58E-08 | 7.66E-07 | 5.68E-05 |
| ***Talaromyces marneffei* C6F_383099** | -9.30 | -629.93 | 2.60E-08 | 7.66E-07 | 5.72E-05 |

^a^Species were selected and arranged based on Log_2_ Fold change
