# Supplementary material for: Candida species and oral mycobiota of patients clinically diagnosed with oral thrush
Source: PLoS One. 2023 Apr 17;18(4):e0284043. doi: 10.1371/journal.pone.0284043 (PMC10109505; doi:10.1371/journal.pone.0284043)
Supplement: S8 Table — (DOCX) [file pone.0284043.s008.docx]

**S8 Table. Significant differential abundance (20 overabundant and 1 underabundant) of fungal strains in AT vs. HC oral rinse samples.**

| **Species^a^** | **Log_2_ Fold change** | **Fold change** | **p-value** | **FDR p-value** | **Bonferroni p-value** |
| --- | --- | --- | --- | --- | --- |
| ***Auricularia cornea*** | 11.13 | 2242.50 | 9.54E-09 | 2.10E-05 | 2.10E-05 |
| ***Candida orthopsilosis*** | 13.27 | 9847.6 | 2.65E-07 | 0.000291 | 5.82E-04 |
| ***Chalastospora gossypii* SH128610.07** | 11.74 | 3409.82 | 5.23E-07 | 0.000383 | 1.15E-03 |
| ***Cladosporium halotolerans*** | 9.32 | 638.63 | 1.89E-06 | 0.000986 | 4.15E-03 |
| ***Coriolopsis strumosa*** | 10.46 | 1404.63 | 2.62E-06 | 0.000986 | 5.76E-03 |
| ***Phaeococcomyces nigricans*** | 10.36 | 1312.46 | 2.69E-06 | 0.000986 | 5.91E-03 |
| ***Exidia pithya*** | 10.09 | 1088.43 | 3.96E-06 | 0.001242 | 8.70E-03 |
| ***Rhodotorula mucilaginosa*** | 10.95 | 1979.03 | 6.77E-06 | 0.001412 | 1.49E-02 |
| ***Chalastospora gossypii* F5F_871543** | 9.93 | 976.74 | 8.22E-06 | 0.001412 | 1.81E-02 |
| ***Neurospora terricola*** | 7.42 | 170.71 | 9.09E-06 | 0.001412 | 2.00E-02 |
| ***Septoria oenanthicola*** | 7.80 | 223.18 | 9.26E-06 | 0.001412 | 2.04E-02 |
| ***Pseudolagarobasidium acaciicola*** | 8.15 | 283.09 | 9.40E-06 | 0.001412 | 2.07E-02 |
| ***Phlebiopsis flavidoalba*** | 9.46 | 705.35 | 1.06E-05 | 0.001412 | 2.33E-02 |
| ***Heterochaete shearii*** | 10.43 | 1383.74 | 1.07E-05 | 0.001412 | 2.35E-02 |
| ***Candida metapsilosis*** | 9.29 | 625.88 | 1.36E-05 | 0.001412 | 2.99E-02 |
| ***Aspergillus waksmanii*** | 9.17 | 574.67 | 1.38E-05 | 0.001412 | 3.04E-02 |
| ***Neofomitella rhodophaea*** | 9.23 | 599.37 | 1.44E-05 | 0.001412 | 3.17E-02 |
| ***Resinicium saccharicola*** | 9.09 | 546.31 | 1.87E-05 | 0.001412 | 4.11E-02 |
| ***Gibellulopsis piscis*** | 9.06 | 533.03 | 2.00E-05 | 0.001412 | 4.40E-02 |
| ***Xeromyces bisporus*** | 9.02 | 519.69 | 2.18E-05 | 0.001412 | 4.79E-02 |
| ***Candida dubliniensis*** | -9.05 | -528.44 | 5.71E-06 | 0.001394 | 1.25E-02 |

^a^Species were selected and arranged based on Log_2_ Fold change
