# Supplementary material for: Candida species and oral mycobiota of patients clinically diagnosed with oral thrush
Source: PLoS One. 2023 Apr 17;18(4):e0284043. doi: 10.1371/journal.pone.0284043 (PMC10109505; doi:10.1371/journal.pone.0284043)
Supplement: S9 Table — (DOCX) [file pone.0284043.s009.docx]

**S9 Table. Significant differential abundance (top 25 overabundant, 2 underabundant) of fungal strains in AT vs. OT oral rinse samples.**

| **Species^a^** | **Log_2_ Fold change** | **Fold change** | **p-value** | **FDR p-value** | **Bonferroni p-value** |
| --- | --- | --- | --- | --- | --- |
| ***Auricularia cornea*** | 13.75 | 13763.23 | 1.27E-14 | 2.78E-11 | 2.78E-11 |
| ***Phaeococcomyces nigricans*** | 11.27 | 2474.12 | 7.50E-12 | 5.84E-09 | 1.65E-08 |
| ***Coriolopsis strumosa*** | 11.37 | 2639.55 | 8.74E-12 | 5.84E-09 | 1.92E-08 |
| ***Exidia pithya*** | 11.02 | 2069.93 | 1.24E-11 | 5.84E-09 | 2.73E-08 |
| ***Chalastospora gossypii* SH128610.07** | 10.76 | 1734.89 | 2.14E-11 | 7.83E-09 | 4.70E-08 |
| ***Phlebiopsis flavidoalba*** | 10.42 | 1370.04 | 5.40E-11 | 1.23E-08 | 1.19E-07 |
| ***Aspergillus waksmanii*** | 10.17 | 1151.54 | 5.59E-11 | 1.23E-08 | 1.23E-07 |
| ***Candida metapsilosis*** | 10.26 | 1223.02 | 7.57E-11 | 1.23E-08 | 1.66E-07 |
| ***Chalastospora gossypii* F5F_871543** | 10.88 | 1886.93 | 7.58E-11 | 1.23E-08 | 1.67E-07 |
| ***Neofomitella rhodophaea* F3F_716953** | 10.20 | 1173.79 | 7.77E-11 | 1.23E-08 | 1.71E-07 |
| ***Neurospora terricola*** | 10.21 | 1186.62 | 7.84E-11 | 1.23E-08 | 1.72E-07 |
| ***Resinicium saccharicola* F3F_720090** | 10.07 | 1074.97 | 1.26E-10 | 1.51E-08 | 2.76E-07 |
| ***Resinicium tenue*** | 9.80 | 892.93 | 1.38E-10 | 1.51E-08 | 3.05E-07 |
| ***Gibellulopsis piscis*** | 10.04 | 1050.18 | 1.42E-10 | 1.51E-08 | 3.12E-07 |
| ***Septoria oenanthicola*** | 10.30 | 1258.64 | 1.44E-10 | 1.51E-08 | 3.17E-07 |
| ***Resinicium saccharicola* SH114229.07** | 9.65 | 802.17 | 1.81E-10 | 1.76E-08 | 3.98E-07 |
| ***Neofomitella rhodophaea* F3F_719155** | 9.89 | 950.66 | 1.84E-10 | 1.76E-08 | 4.05E-07 |
| ***Xeromyces bisporus*** | 9.34 | 648.51 | 2.47E-10 | 2.09E-08 | 5.42E-07 |
| ***Ganoderma australe*** | 9.41 | 678.46 | 4.02E-10 | 2.91E-08 | 8.841E-07 |
| ***Ilyonectria macrodidyma*** | 8.63 | 395.04 | 4.45E-10 | 2.91E-08 | 9.77E-07 |
| ***Trichoderma asperellum*** | 8.47 | 355.07 | 8.74E-10 | 5.06E-08 | 1.92E-06 |
| ***Polyporus rugulosus*** | 10.19 | 1168.04 | 9.91E-10 | 5.59E-08 | 2.18E-06 |
| ***Didymella calidophila*** | 8.91 | 482.31 | 1.06E-09 | 5.63E-08 | 2.34E-06 |
| ***Lentinus squarrosulus*** | 9.15 | 566.81 | 1.09E-09 | 5.63E-08 | 2.41E-06 |
| ***Macrocybe gigantea*** | 9.40 | 673.31 | 1.12E-09 | 5.63E-08 | 2.46E-06 |
| ***Candida dubliniensis* G8F_551382** | -8.61 | -389.39 | 4.05E-07 | 4.46E-06 | 8.91E-04 |
| ***Candida dubliniensis* SH101749.07** | -11.18 | -2322.67 | 2.14E-06 | 1.60E-05 | 4.71E-03 |

^a^Species were selected and arranged based on Log_2_ Fold change
