# Supplementary material for: Candida species and oral mycobiota of patients clinically diagnosed with oral thrush
Source: PLoS One. 2023 Apr 17;18(4):e0284043. doi: 10.1371/journal.pone.0284043 (PMC10109505; doi:10.1371/journal.pone.0284043)
Supplement: S10 Table — (DOCX) [file pone.0284043.s010.docx]

**S10 Table. Alpha diversity index values for OT, HC and AT groups.**

| **Index** | **Oral thrush (OT) n=16** | | **Healthy control (HC)**  **n=7** | | **Follow-up (AT)**  **n=7** | | **Kruskal-Wallis p-value** |
| --- | --- | --- | --- | --- | --- | --- | --- |
|  | **Median** | **IQR^a^** | **Median** | **IQR^a^** | **Median** | **IQR^a^** |  |
| Shannon | 2.13 | 5.08 | 5.08 | 2.50 | 2.65 | 4.10 | 0.03* |
| Chao-1 biased corrected | 304.23 | 498.15 | 498.15 | 207.45 | 422.21 | 172.33 | 0.01* |

^a^Abbreviations: IQR, interquartile range

*p < 0.05, statistically significant
